# Early evolution of the LIM homeobox gene family

**DOI:** 10.1186/1741-7007-8-4

**Published:** 2010-01-18

**Authors:** Mansi Srivastava, Claire Larroux, Daniel R Lu, Kareshma Mohanty, Jarrod Chapman, Bernard M Degnan, Daniel S Rokhsar

**Affiliations:** 1 Center for Integrative Genomics and Department of Molecular and Cell Biology, University of California, Berkeley, CA, USA; 2 School of Biological Sciences, The University of Queensland, Brisbane, Queensland, Australia; 3 Department of Energy, Joint Genome Institute, Walnut Creek, CA, USA

## Abstract

**Background:**

LIM homeobox (Lhx) transcription factors are unique to the animal lineage and have patterning roles during embryonic development in flies, nematodes and vertebrates, with a conserved role in specifying neuronal identity. Though genes of this family have been reported in a sponge and a cnidarian, the expression patterns and functions of the Lhx family during development in non-bilaterian phyla are not known.

**Results:**

We identified *Lhx *genes in two cnidarians and a placozoan and report the expression of *Lhx *genes during embryonic development in *Nematostella *and the demosponge *Amphimedon. *Members of the six major LIM homeobox subfamilies are represented in the genomes of the starlet sea anemone, *Nematostella vectensis*, and the placozoan *Trichoplax adhaerens*. The hydrozoan cnidarian, *Hydra magnipapillata*, has retained four of the six Lhx subfamilies, but apparently lost two others. Only three subfamilies are represented in the haplosclerid demosponge *Amphimedon queenslandica. *A tandem cluster of three *Lhx *genes of different subfamilies and a gene containing two LIM domains in the genome of *T. adhaerens *(an animal without any neurons) indicates that Lhx subfamilies were generated by tandem duplication. This tandem cluster in *Trichoplax *is likely a remnant of the original chromosomal context in which Lhx subfamilies first appeared. Three of the six *Trichoplax Lhx *genes are expressed in animals in laboratory culture, as are all *Lhx *genes in *Hydra*. Expression patterns of *Nematostella Lhx *genes correlate with neural territories in larval and juvenile polyp stages. In the aneural demosponge, *A. queenslandica*, the three *Lhx *genes are expressed widely during development, including in cells that are associated with the larval photosensory ring.

**Conclusions:**

The Lhx family expanded and diversified early in animal evolution, with all six subfamilies already diverged prior to the cnidarian-placozoan-bilaterian last common ancestor. In *Nematostella*, *Lhx *gene expression is correlated with neural territories in larval and juvenile polyp stages. This pattern is consistent with a possible role in patterning the *Nematostella *nervous system. We propose a scenario in which *Lhx *genes play a homologous role in neural patterning across eumetazoans.

## Background

In contrast to the centralized and highly structured nervous systems of bilaterians, some animals (cnidarians and ctenophores) have more simply organized networks, and still others (sponges and placozoans) appear to lack a nervous system entirely [1]. To the extent that these early branching animal phyla (the so called 'basal metazoa') have retained early metazoan characters, their study can inform our understanding of the early evolution of the nervous system. Although early metazoan phylogeny remains controversial [2-5], among the living phyla sponges were likely the first animal group to diverge, followed by the subsequent branching of placozoans, and then cnidarians/bilaterians. (The placement of ctenophores remains contentious [3,6]). Both sponges [7] and placozoans (that is, *Trichoplax adhaerens*) [8] appear to lack a defined neuronal cell type, although evidence for putative sponge neurons has been put forward [9], and the genes corresponding to postsynaptic scaffolding have been identified in a demosponge [10]. In contrast, cnidarians (hydra, anemones, corals, jellyfish) all have clearly defined neurons [11], and neural networks of varying complexity (see, for example, [12-20]). The differences between early branching phyla are traditionally thought to represent the evolutionary progression of the nervous system in the first animals, but molecular evidence supporting such gradual evolution has been lacking. Comparative analysis of nervous system patterning genes in diverse animal phyla with and without nervous systems provides an avenue for understanding the early evolution of this fundamental animal feature.

Genes of the LIM homeobox (Lhx) family perform fundamental roles in tissue-specific differentiation and body patterning during development in both vertebrates and invertebrates [21,22] (summarized in Additional file 1, Table S1). These genes comprise a family of DNA-binding proteins with six subfamilies; each subfamily member is represented once in *Caenorhabditis elegans *and *Drosophila melanogaster *and twice in mammalian species [23]. Lhx proteins are composed of two N-terminal LIM domains (named after the founding members LIN-11, Islet-1, and MEC-3) and a helix-turn-helix forming homeodomain that binds regulatory DNA surrounding target genes [22,24]. The zinc-finger forming LIM domains are essential for protein function in several subfamilies and are thought to regulate DNA binding by the homeodomain by interacting with other nuclear proteins [23]. The diverse functions of Lhx proteins include the development of kidney, pancreas, eyes, and limbs in vertebrates (by the Lhx1/5, Lhx3/4, Islet, Apterous, and Lmx subfamilies), the patterning of wings and imaginal disc precursor tissues in flies (by Apterous and Arrowhead), and the formation of the vulva in *C. elegans *(LIN-11 or Lhx1/5 family) [23]. *Lhx *genes mediate these developmental functions by specifying cellular identities and their loss of function can result in human disease [25,26].

While Lhx proteins perform a diverse array of developmental functions, all members of the Lhx family are prominent in specifying the fates of motorneurons, sensory neurons, and interneurons [23]. More specifically, in both vertebrates and *Drosophila*, motorneuron subtype identity is determined by a combinatorial code of *Lhx *genes and a particular *Lhx *gene defines interneuron subtype identity, suggesting that these genes played such roles in the common ancestor of bilaterians [23,27-29]. Lmx proteins specify serotonergic neurons [30,31]; *Lmx *genes are also implicated in dopaminergic neural fates [32]; *Lhx8 *and *islet *specify cholinergic fate [33-37]; GABAergic fates are specified by *Lhx7 *and *Lhx6 *[36,38,39]. Many *Lhx *genes are involved in the development of various types of sensory neurons, such as photosensory, thermosensory, olfactory, chemosensory, or mechanosensory neurons (see, for example, [40-44]).

Classic studies in *Hydra*, a hydrozoan cnidarian, and other cnidarians showed that the adult nervous system is composed of regionalized and overlapping populations of cells expressing various neurotransmitters and neuropeptides [12-19]. Recently, the anatomy of the nervous system over developmental time has been studied in the anthozoan starlet sea anemone, *Nematostella vectensis *[20], revealing neural complexity comparable to that of *Hydra*. Are cnidarian neuronal subpopulations patterned in a manner similar to those in bilaterians, for example, using combinatorial expression of *Lhx *genes? If so, are these patterning mechanisms in place in placozoans and sponges despite the lack of nervous systems in these phyla?

LIM homeobox genes have been reported in the genomes of *N. vectensis *[45] and the demosponge *Amphimedon queenslandica *[46,47]. Using the recently sequenced genomes of *N. vectensis *[48], *Hydra magnipapillata *[49], *T. adhaerens *[2], and *A. queenslandica *(Srivastava *et al.*: The genome of the haplosclerid demosponge *Amphimedon queenslandica *and the evolution of animal complexity, submitted), we trace the evolution of the LIM homeobox family. We then report the expression patterns of several *Lhx *gene families during embryonic development in *N. vectensis *and *A. queenslandica*. The territories of expression of these genes broadly overlap those of known neuronal subpopulations in the sea anemone, and putative photosensory cells in the sponge.

## Results

### Origin and early diversification of the LIM homeobox protein family

Genes with the LIM-LIM homeobox domain composition were found in all the animal genomes queried in this study. However, no putative Lhx proteins were predicted in the genome of *Monosiga brevicollis*, a unicellular choanoflagellate (the sister group to animals). This, together with the absence of LIM-LIM homeobox proteins in plants, fungi and other eukaryotes suggests that the combination of LIM domains and homeodomains is unique to animals.

**Figure 1:**
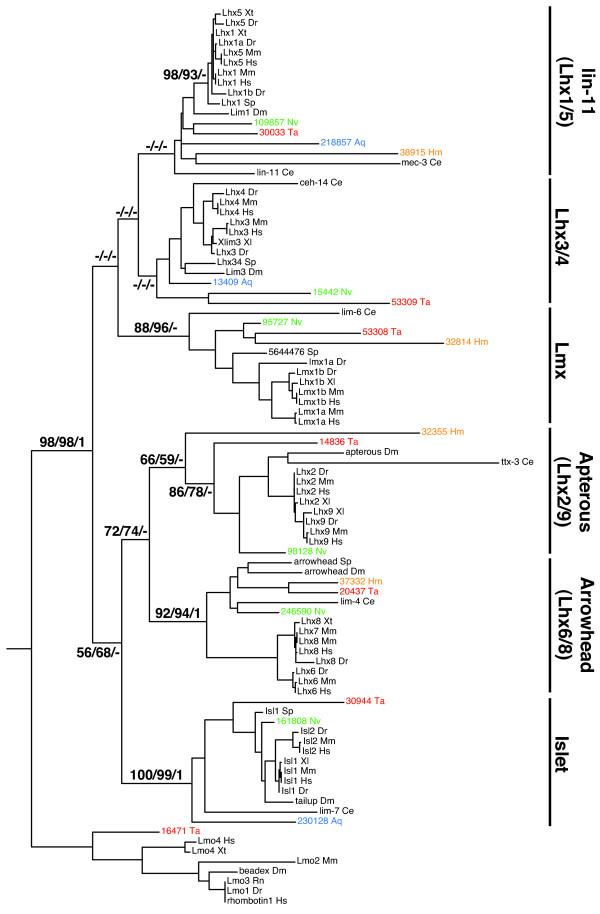
**Phylogeny of LIM homeobox genes**. The maximum likelihood tree based on an alignment of two LIM domains and the homeodomain is shown here with support values from Neighbor-joining/Likelihood/Bayesian analyses shown for the major nodes (relationships within the major classes were well supported only for vertebrate sequences). Neighbor-joining and Likelihood bootstrap values above 50% are shown, as are Bayesian posterior probabilities above 0.95. Full trees from each analysis are shown in Additional file 1. Aq = *Amphimedon queenslandica*(blue); Ce = *Caenorhabditis elegans*; Dm = *Drosophila melanogaster*; Dr = *Danio rerio*; Hm = *Hydra magnipapillata *(orange); Hs = *Homo sapiens*; Nv = *Nematostella vectensis *(green); Rn = *Rattus norvegicus*; Sp = *Strongylocentrotus purpuratus*; Ta = *Trichoplax adhaerens *(red); Xt = *Xenopus tropicalis*.

The *Nematostella *genome encodes six Lhx proteins, which each fall into one of the six known subfamilies (Figure 1). In addition to the three *Lhx *genes classified into Islet, Lhx1/5 (LIN-11), and Lhx6/8 (Arrowhead) groups previously [45], we identified orthologs of the Lhx3/4, Lhx2/9 (Apterous) and Lmx groups in *Nematostella *(as found in [47]). The *Lmx *gene appears to have only one LIM domain, contrary to the usual two LIM domains followed by a homeodomain composition known from bilaterian Lhx genes (Table 1). As is the case with *Nematostella*, members of all six Lhx subfamilies are represented in the *Trichoplax *genome. This implies that the six Lhx subfamilies were already established in the common ancestor of cnidarians, placozoans, and bilaterians. While the putative *Trichoplax *Lhx6/8 (Arrowhead) ortholog encodes only two LIM domains but no homeodomain, it can nevertheless be robustly classified as a member of the Arrowhead/Lhx6/8 subfamily.

**Table 1:** Summary of domain structures and expression evidence for LIM homeobox genes found in four early animal genomes

Subfamily	*Nematostella*		*Hydra*		*Trichoplax*		*Amphimedon*	
	
	Domains	Expressed	Domains	Expressed	Domains	Expressed	Domains	Expressed
Apterous (Lhx2/9)	L-L-H	Y	L-L-H	Y	L-L-H	Y	-	-

Arrowhead (Lhx6/8)	L-L-H	Y	L-H	Y	L-L	Y	-	-

Islet	L-L-H	Y	-	-	L-L-H	-	L-L-H	Y

lin-11 (Lhx1/5)	L-L-H	Y	L-L-H	Y	L-L-H	Y	L-L-H	Y

Lhx3/4	L-L-H	N	-	-	L-L-H	-	L-L-H	Y

Lmx	L-H	Y	L-L-H	Y	L-L-H	-	-	-

H = homeodomain; L = LIM domain; N = not found to be expressed by reverse transcription polymerase chain reaction (RT-PCR); Y = expressed.

Only four *Lhx *genes were identified in *Hydra*, each orthologous to a different Lhx subfamily (Arrowhead, Apterous, Lmx, Lhx1/5) suggesting that members of the other subfamilies (Islet, Lhx3/4) have been lost along the lineage leading to *Hydra*, after its divergence from anthozoans (Figure 1). The *Hydra *member of the Arrowhead subfamily appears to be missing the first LIM domain (Table 1). The *Amphimedon *complement of Lhx proteins consists of members of the Islet, Lhx3/4 and Lhx1/5 families [46,47]. Given the poor support for the relationships of Lhx subfamilies to each other, we cannot distinguish between two scenarios: first, that three Lhx subfamilies were lost in the *Amphimedon *lineage, and second, that the common ancestor of all animals may have only had three Lhx genes, with ancestral (and sponge) genes most resembling specific daughter families because of asymmetric evolutionary rates of gene duplicates [47].

Though there is poor support in the tree (Figure 1) for the Lhx1/5 and Lhx3/4 groups, *Nematostella*, *Trichoplax*, *Hydra*, and *Amphimedon *genes have been assigned to these subfamilies because these classifications are the most likely scenario. It is often difficult to classify genes from early-branching animal phyla into clear bilaterian subfamilies [47,50] and the inability to find good bootstrap support for the Lhx1/5 and Lhx3/4 groups may be a result of high levels of divergence between the early animal sequences relative to their bilaterian counterparts. Indeed, in an Lhx tree constructed without *Trichoplax *or *Hydra *sequences, assignment of *Nematostella *and *Amphimedon *genes to specific subfamilies was well supported [47]. Also, given that *Nematostella *and *Trichoplax *have genes that can be confidently placed in each one of the other subfamilies (Arrowhead, Islet, Apterous, Lmx), it is likely that the tree in Figure 1 has recovered the correct placements of the *Nematostella *and *Trichoplax *Lhx1/5 and Lhx3/4 proteins.

### Synteny and intron conservation of *Lhx *genes

**Figure 2:**
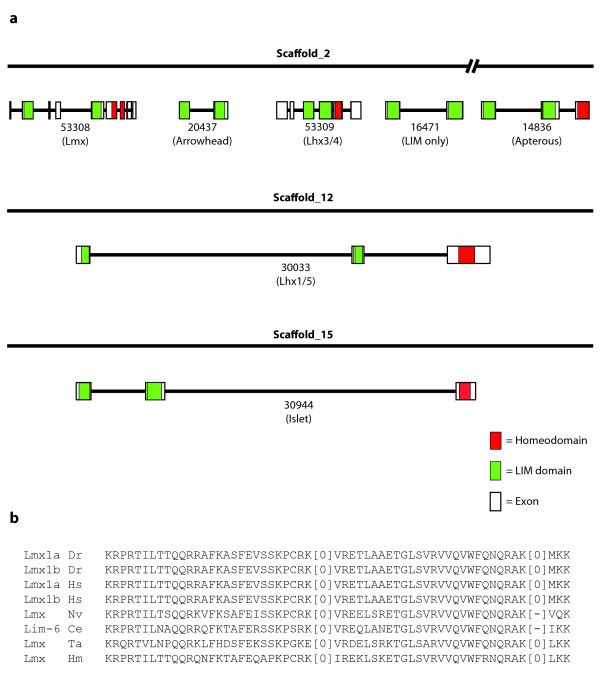
**Synteny and intron conservation of LIM homeobox genes**. **(a) **Four of the six *Trichoplax *LIM homeobox genes are present on one scaffold, three of these are present in tandem. This tandem cluster also contains a gene coding for a protein of the LIM only (Lmo) class. This scaffold is in the same putative ancestral linkage group as human chromosome segments that contain 6 of the 12 human LIM homeobox genes. **(b) **Two introns that interrupt the homeodomain in the Lmx class proteins are well conserved across animals, but one has been lost in both *Nematostella *and *C. elegans*. Introns are represented with square brackets with the enclosed number indicating the phase of the intron.

Of the six putative *Lhx *genes in the *Trichoplax *genome, three are present as part of a tandem cluster on scaffold_2 that also includes another LIM-LIM domain containing gene (Figure 2a) (Additional file 1, Supplemental Section 2). This fourth member of the tandem cluster can be classified as a member of the Lmo family using phylogenetic methods (Figure 1). The three *Lhx *genes in the cluster belong to the Lmx, Arrowhead and Lhx3/4 subfamilies, and a fourth *Lhx *gene (of the Apterous subfamily) is present further downstream on scaffold_2. The classification of these proteins into distinct Lhx subfamilies suggests that these syntenic genes are unlikely to be the result of a recent duplication in the placozoan lineage. Therefore, this syntenic cluster of genes likely represents (that is, is a remnant of) the ancestral genomic context in which the different Lhx subfamilies first evolved. The preservation of this tandem cluster in *Trichoplax *(with only three Lhx families missing) and its disruption in most other genomes is consistent with the finding that the *Trichoplax *genome appears to be the least rearranged relative to the inferred ancestral genome [2]. Of the 12 *Lhx *genes in humans, 7 are located on segments in different human chromosomes, but these segments fall into the same ancestral linkage groups as the tandem cluster of *Trichoplax Lhx *genes (Additional file 1, Table S10) [51]. This suggests that the tandem *Lhx *gene cluster in *Trichoplax *descended from the same ancestral genomic context that gave rise to modern bilaterian *Lhx *genes.

Introns are found in diverse bilaterian homeobox-containing genes at over 20 different positions that interrupt the homeobox [52]. In the bilaterian members of the Lmx subfamily, the homeodomains are interrupted by two conserved introns (Figure 2b). The first of these introns is found to be present in the cnidarian and placozoan orthologs of Lmx as well, but the second one has been lost in *Nematostella *(though it is present in *Hydra *and *Trichoplax Lmx *genes).

### Normal and atypical *Lhx *genes are expressed in *Nematostella*, *Hydra*, *Trichoplax*, and *Amphimedon*

All four *Hydra Lhx *genes were successfully amplified from the cDNA of adults (some of which may have been reproducing asexually) (Table 1). The *apterous *gene model in *Hydra *was found to be incorrect as 5' rapid amplification of complementary DNA ends (RACE) determined the expression of another exon containing the second LIM domain that was found to be encoded in the genomic sequence upstream of the predicted gene model. However, 5' RACE verified that the *Hydra arrowhead *gene model, which also predicted only one LIM domain, is correct. Lmx and Lhx1/5 orthologs in *Hydra *also appeared to be missing a LIM domain, but lowering the e-value threshold in the National Center for Biotechnology Information (NCBI) Conserved Domain Search tool [53] identified an additional N-terminal LIM domain in the *Hydra *Lmx gene model and an additional C-terminal LIM domain in the *Hydra *Lhx 1/5 prediction. The expression of both LIM domains and the homedomain in the *Lmx *and *Lhx1/5 *orthologs was confirmed through molecular cloning and sequencing analysis. These findings suggest that *Hydra *Lhx protein LIM domains have an accelerated rate of evolution (resulting in decreased affinity to the position specific weight matrices that define conserved domains), consistent with the overall high rate of protein sequence evolution in the *Hydra *lineage.

Of the six *Trichoplax Lhx *orthologs, *apterous*, *arrowhead *and *Lhx1/5 *were found to be expressed in the animals in laboratory culture (The *Lmo *gene in the tandem cluster on scaffold_2 is expressed as well). Five of the six *Nematostella *genes were also amplified from cDNA of animals at various developmental stages, including the Lmx-like gene that is missing one LIM domain (Table 1). Thus, *Lhx *genes with atypical domain compositions predicted in the genomes of *Nematostella*, *Trichoplax *and *Hydra*, are found to be expressed in those configurations (no atypical forms were found in *Amphimedon*). This finding is similar to those in other families such as the Hedgehog ligand, where early animal phyla are found to encode proteins with domain compositions not seen in homologous sequences in bilaterians [54,55]. However, since these configurations are not shared between phyla (for example, *Hydra *and *Trichoplax *Arrowhead proteins have different missing domains), they most likely resulted from independent evolution along these lineages.

### *Nematostella Lhx *genes are expressed in discrete regions of developing embryos

**Figure 3:**
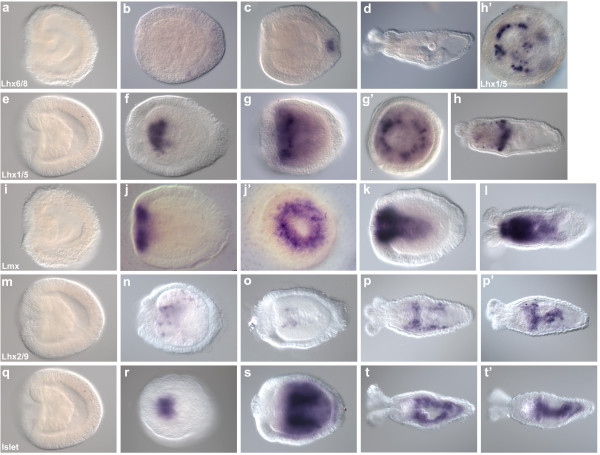
**LIM homeobox gene expression during *Nematostella *development**. **(a-d) **The *arrowhead *(*Lhx6/8*) ortholog is first expressed in the apical tuft of the late planula (c) but disappears in the juvenile polyp (d). **(e-h) **The *lin-11 *(*Lhx1/5*) ortholog is first expressed in the putative pharyngeal endoderm in the early planula and later resolves into an endodermal ring around the pharynx (g' = oral view of g; h' = cross-section through h). **(i-l) **The *Lmx *ortholog is first transcribed in the oral ectoderm of the early planula and then spreads into the pharynx and directive mesenteries (l' = oral view of l). **(m-p) **The *apterous*(*Lhx2/9*) ortholog is expressed in the planula endoderm in a speckled pattern and later its expression spreads to the end of the pharynx and throughout the directive mesenteries (p' = lateral view of p). **(q-t) **The *islet *ortholog starts out in the putative pharyngeal endoderm and over time spreads into the directive mesenteries. This gene is transcribed in cells of the planula body wall endoderm and in the polyp stage there it shows restricted expression in the aboral endoderm (t' = lateral view of t).

The mRNA for the *Lhx6/8 *(*arrowhead*) ortholog in *Nematostella *first appears faintly in the aboral ectoderm in the early planula and subsequently its expression resolves to mark ectodermal cells in the apical tuft in late planula stages (Figure 3a-c). This mRNA is absent in juvenile polyp stages (Figure 3d). *Lhx1/5 *(*lin-11*) expression in *Nematostella *begins in the early planula in endoderm cells that will form the endoderm around the pharynx (Figure 3e-g). This expression persists in late planula and juvenile polyp stages in discrete clusters of cells in a ring around the pharyngeal endoderm (Figure 3g,h). The expression of this gene around the pharynx appears to be radial, with no apparent asymmetries (Figure 3h'). A third *Lhx *gene, the *Lmx *ortholog, starts out with strong expression in the oral ectoderm in the early planula, and over time its expression spreads to the pharynx and the endodermal tissue that will make the directive mesenteries (that is, the pair of endodermal infoldings that are the first to appear) (Figure 3i-k). In juvenile polyps, *Lmx *mRNA has strong expression in the pharyngeal endoderm and ectoderm and weak expression in the directive mesenteries (Figure 3l). The *Lhx2/9 *(*apterous*) ortholog in *Nematostella *has speckled expression throughout the endoderm in early planula stages, but is found in a few cells of the aboral region of the pharynx in the late planula stage (Figure 3m-o). Juvenile polyps express *Lhx2/9 *in the aboral end of the pharynx and in the directive mesenteries (Figure 3p,p'). The *islet *gene of *Nematostella *is expressed in the pharynx as it starts to form and its mRNA is found in directive mesenteries and aboral endoderm of later stages (Figure 3q-t).

### *Amphimedon *Lhx genes are expressed during embryogenesis

**Figure 4:**
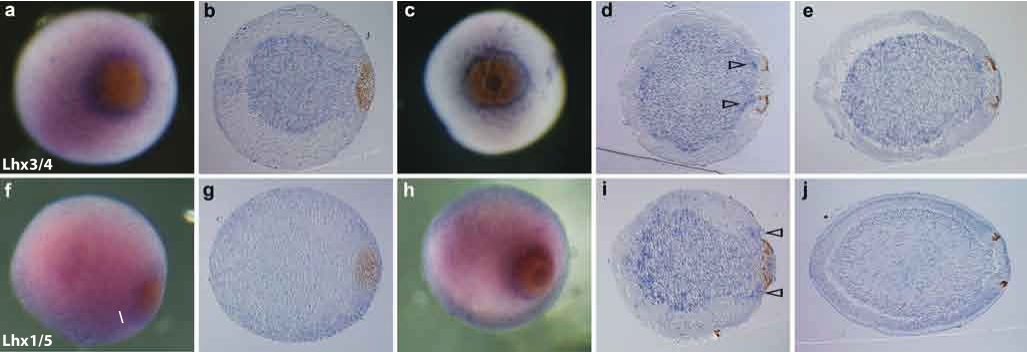
**LIM homeobox gene expression during *Amphimedon *development**. **(a, c, f, h) **Whole-mount micrographs; **(b, d, e, g, i, j) **micrographs of sectioned embryos. (a-e) The *Lhx3/4 *ortholog is expressed in the inner cell mass during late gastrulation, when pigment cells form a spot (a,b) and then a ring (c,d). A stronger expression domain appears transiently under the photoreceptor ring when it is forming (arrowheads in d). Expression is ubiquitous in the prehatched larva, with higher expression levels in the subepithelial layer (e). (f-j) The *Lhx1/5 *ortholog appears to be expressed in the outer layer at the pigment spot stage, especially around the spot (f,g). When the pigment ring forms (h,i), the gene is highly expressed in the inner cell mass, especially inside the developing ring and at the anterior end. A strong expression domain also appears in the micromeres surrounding the developing pigment ring (arrowheads in i). Expression seems to be ubiquitous in the larva before it hatches (j).

In *Amphimedon*, *Lhx3/4 *(*lim-3*) is expressed in the inner cell mass with transiently higher expression levels under the photoreceptor pigment ring as it develops (Figure 4a-e). When pigment cells have coalesced into a spot, *Lhx1/5 *(*lin-11*) appears to be expressed in the outer cell layer of the embryo, with higher levels of expression observed in cells around the pigment spot (Figure 4f,g). *Lhx1/5 *expression remains associated with the pigment ring as it forms and dramatically increases in the inner cell mass, especially at the anterior end (Figure 4h,i). Both of these genes appear to be ubiquitously expressed in a relatively uniform manner in the larva just prior to hatching (Figure 4e,j). The *islet *gene appears to be ubiquitously expressed during *Amphimedon *development (data not shown).

## Discussion

The six Lhx subfamilies originally identified in flies, nematodes, and vertebrates are all represented in the *Trichoplax *and *Nematostella *genomes, indicating that the diversification of the Lhx family by gene duplication had already occurred by the time of the last common bilaterian-cnidarian-placozoan ancestor. In *Trichoplax*, four of the six *Lhx *genes are colocalized to a region of a few hundred kb in the genome. This implies that the diversification of the Lhx family took place by tandem (or local) gene duplication, and that some of these linkages have been retained in the *Trichoplax *lineage. This is analogous to the diversification of the Hox cluster, which arose by tandem duplication in the bilaterian lineage and is preserved in multiple extant lineages. For the Lhx cluster, only *Trichoplax *preserves remnants of the ancestral organization.

The *Amphimedon *genome contains three Lhx subfamily members (Lhx1/5, Lhx3/4, and Islet) but we cannot resolve whether these three represent the ancestral metazoan Lhx complement, with Lmx, Arrowhead, and Apterous arising by duplication from within these families in the placozoan-cnidarian-bilaterian lineage, or if the sponge lost these subfamilies. Interestingly, the three Lhx gene subfamilies found on the same scaffold as the *Lhx3/4 *gene in the placozoan genome are missing from the sponge genome. From the phylogenetic tree, we cannot reject the possibility that these three genes arose after the divergence of sponges, from an initial duplication of the *Lhx3/4 *gene. Analysis of *Lhx *genes in other sponges may resolve this issue.

In contrast to *Trichoplax *and *Nematostella*, the *Hydra *genome lacks members of the Lhx3/4 and Islet subfamilies, which were evidently lost in the *Hydra *lineage. The *arrowhead *gene in *Hydra *has an atypical structure, lacking one of the two LIM domains characteristic of the family. Although such domain structures have not been reported in bilaterians, independently evolved atypical domain structures are also observed in *Nematostella *and *Trichoplax Lhx *genes. Nevertheless, such genes are expressed, suggesting that they retain some function and are not simply pseudogenes. Some Lhx proteins show long branch lengths on phylogenetic trees, suggesting that these subfamily members may be experiencing reduced constraint and/or positive selection.

**Figure 5:**
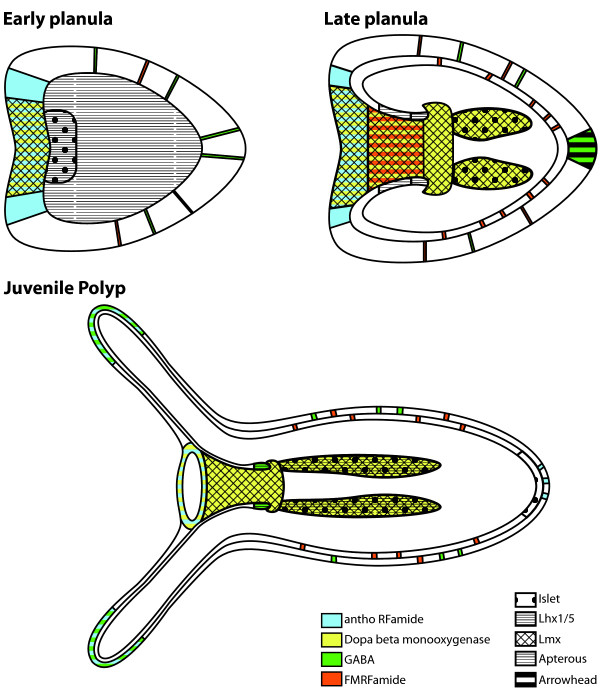
**Schematic diagrams of *Nematostella *developmental stages showing combinatorial expression of LIM homeobox genes and overlap with known functionally different neural types**. Neurons with putatively different functions emerge over the course of embryonic development (as assayed by neurotransmitter antibodies and *in situ *hybridization to detect neuropeptide or neurotransmitter synthesis enzyme mRNA) [20]. LIM homeobox genes have dynamic expression patterns that overlap with each other, as well as with territories of different neural types. The oral nerve ring (marked by 3,4-dihydroxyphenylalanine (DOPA- β-monoxygenase and RFamide), the pharyngeal nerve ring (marked by ©-aminobutyric acid (GABA)) and the apical tuft (marked by GABA) correspond to *Lmx*, *Lhx1/5 *and *arrowhead *expression respectively. DOPA-β-monoxygenase expression over developmental time is mirrored by *Lmx *expression. Two-color stripes show expression of two neural markers in the same region.

In diverse bilaterians, the LIM homeobox 'code' is conserved in the sense that neural types are patterned by combinatorial expression of *Lhx *and other transcription factors; however, the same types are not generated by the same *Lhx *combinations in different species [23]. In *Nematostella*, the expression of *Lhx *genes during embryonic development also appears to correlate with neural territories, although we have not shown that these genes are expressed in neurons. Three different LIM homeobox genes are expressed in the three major neuralized regions: the apical tuft of the planula, and the oral and pharyngeal nerve rings in the polyp (Figure 5) [20]. DOPA-β-monoxygenase, the enzyme involved in epinephrine and norepinephrine synthesis, and anthoRFamide mRNA mark the oral nerve ring, a region that is found to express the *Nematostella Lmx *ortholog. Over the course of development, *Lmx *expression spreads into the pharynx and directive mesenteries, mirroring the changes in DOPA-β-monoxygenase expression. The *Lhx6/8 *(*arrowhead*) ortholog is expressed transiently in the apical tuft, a region found to have GABAergic sensory cells. The *Lhx1/5 *ortholog marks clusters of cells in an endodermal ring at the end of the pharynx, a region that contains a ring of GABA-positive neurons. In a recent paper, Yasuoka *et al*. [56] found that this gene is expressed around the blastopore during gastrulation, and suggested that this gene had an ancestral role as a blastoporal organizer. However, we have not detected any *Nematostella Lhx1/5 *expression at this stage.

Our data provide circumstantial evidence supporting the hypothesis that *Lhx *genes in *Nematostella *are involved in combinatorially specifying neuronal identity, as they are in bilaterians, based on the coincidence of *Lhx *expression territories and regions where distinct neural populations are found. The hypothesis predicts that *Lhx *genes should be expressed in neurons themselves, which has yet to be shown. There are other predictions. For example, although only one functional neural type (adrenergic) has been found in *Nematostella *mesenteries thus far, based on the combinatorial expression of *islet*, *apterous *and *Lmx *in this specialized endodermal tissue we predict that there should be functional differentiation of neurons in this region relative to the pharynx, which has only *Lmx *expression. Though only the *Lmx *mRNA is found in the oral nerve ring and pharynx, the oral nerve ring contains both RFamide-producing and adrenergic neurons, and a part of the pharynx contains both adrenergic and FMRFamide-expressing neurons. Evidently *Lhx *gene expression is not necessary for neuronal specification in *Nematostella*, however, since none of the five *Lhx *genes assayed here have been found to be expressed in tentacles, although tentacle tips contain spirocysts, GABAergic, and RFamide-expressing cells. Thus, it is likely that other transcription factors in addition to *Lhx *genes are involved in cospecifying functionally different neurons in *Nematostella *(as is the case with the specification of bilaterian neuronal identity). Indeed, other transcription factors are known to be expressed in the neural territories where cnidarian *Lhx *genes are found. For example, *PaxB *(orthologous to bilaterian *Pax2/5/8*) is expressed in an endodermal ring at the base of the pharynx [57], corresponding to the location of the pharyngeal nerve ring.

Similar comparisons of the expression of Lhx family members and the many documented neural populations in *Hydra *[16-19] will be invaluable in understanding the evolution of neural patterning mechanisms. In this study, we found that the four *Lhx *genes encoded by the *Hydra *genome are expressed in adults. We did find that all six *Trichoplax *Lhx subfamilies are present in the genome, and that three subfamilies are expressed in animals in laboratory cultures (Table 1). *Trichoplax *notably has no described nervous system, and only four to five recognized cell types [8,58]. Further characterization of *Lhx *genes in *Trichoplax *could illuminate the ancestral function of these genes, or alternate derived functions if the placozoan-cnidarian-bilaterian ancestor had a nervous system that was lost in the placozoan lineage.

Our observation of patterned expression of Lhx1/5 and Lhx3/4 subfamily members during *Amphimedon *larval development is consistent with a scenario in which Lhx subfamily members were expressed in defined territories in the last common metazoan ancestor. Although *Amphimedon *has no defined neurons, we do observe correlation between *Lhx *gene expression and sensory cells. Both *Lhx1/5 *and *Lhx3/4 *are expressed around the larval pigment ring where photosensory cells form. As the two genes are highly expressed in different but overlapping territories in this region, it is tempting to speculate that the sponge *Lhx *genes are specifying cell identity in a combinatorial manner, as in bilaterians animals. If we further assume that the nervous system is a eumetazoan synapomorphy, originating after the divergence of sponge and placozoan lineages, this hypothesis would imply that the ancestral repertoire of three to six metazoan *Lhx *genes was co-opted into differentiating neural cell types in the first simple nervous systems. Although inferring the original role for these genes in the very first metazoans is difficult, gene expression patterns in *Amphimedon *suggest a number of possibilities, including in the development of non-neural sensory cells. The hypothesized linkage between *Lhx *gene expression and nervous system patterning does not exclude other roles for these genes in early metazoans. For example, shared expression of *Lhx1/5 *in bilaterian gastrulation, the cnidarian blastopore [56], and the sponge pigment spot suggests a possible organizing role during development. Likewise, the expression of *Lhx3/4 *in protochordate endoderm [59,60] and *Amphimedon *inner cell mass points to a potential ancestral role in germ layer formation.

## Conclusions

Through sequence analysis we have shown that the Lhx transcription factor family was already established, and had duplicated and diversified, in the last common metazoan ancestor. We find that *Lhx *genes are expressed in defined, overlapping territories in the sea anemone *Nematostella*. Combined with (1) the neural differentiation observed in these regions and (2) the well established role of *Lhx *genes in the combinatorial control of neural identity in bilaterians, this observation further suggests the hypothesis that *Lhx *genes may play a homologous role in specifying neural identify in non-bilaterians. In this scenario *Lhx *gene expression would be causally linked to the structure of the cnidarian nerve net, whose complexity has been long established in *Hydra *[12-19] and more recently in *Nematostella *[20]. Alternately, the Lhx-neural identity linkage is a bilaterian synapomorphy, and our observed correlations reflect convergent evolution and/or non-homologous processes of neural specification in cnidarians and bilaterians. Early branching animal lineages share a large repertoire of patterning genes with bilaterians, but lack the overt bilaterian differentiation of body axes. We hypothesize that the genes function in defining the molecularly distinguished cell types that various studies are beginning to recognize in cnidarians and sponges [10,20,61,62].

## Methods

### Animal culture, RNA extraction and cDNA synthesis

*N. vectensis *adults (descendents of the CH2 and CH6 cross) were maintained and spawned as described in Fritzenwanker and Technau [63]. *H. magnipapillata *were cultured in *Hydra *medium, consisting of 1% seawater. *T. adhaerens *of the Grell strain collected in the Red Sea were cultured in bowls or Petri dishes filled with filtered artificial seawater at room temperature. Every 2 weeks the bowls were fed with 3 to 5 ml of *Rhodomonas salinas *and salinity and pH were maintained between 32 ppt (parts per thousand) to 35 ppt and 8.0 to 8.4, respectively.

*Nematostella *embryos (collected at various time points), *Hydra *adults (including animals that were undergoing the process of budding) and *Trichoplax *from laboratory cultures (animals were starved for 24 h before collection) were collected, frozen in liquid nitrogen, and stored at -80°C. RNA was then extracted using standard TRIzol (Invitrogen, Carlsbad, USA) protocol. cDNA was made using the Superscript III First-Strand Synthesis System (Invitrogen, Carlsbad, USA) for reverse transcription polymerase chain reaction (RT-PCR) kit. cDNA for 5' and 3' RACE was prepared using the FirstChoice RLM-RACE Kit (Ambion, Austin, USA).

*Amphimedon *embryo and larval collection, RNA extraction, and cDNA synthesis were performed as previously described [64].

### Identification of LIM homeobox genes in cnidarians, placozoans and sponges

Several known LIM homeobox (Lhx) sequences from human, mouse and *D. melanogaster *genomes were aligned using BLAST against the predicted gene models for the genomes of *N. vectensis *http://jgi.doe.gov/nematostella[48], *H. magnipapillata *http://hydrazome.metazome.net/cgi-bin/gbrowse/hydra[49], *T. adhaerens *http://jgi.doe.gov/trichoplax[2], *A. queenslandica *(Srivastava *et al.*: The genome of the haplosclerid demosponge *Amphimedon queenslandica *and the evolution of animal complexity, submitted) and *M. brevicollis *http://jgi.doe.gov/monosiga[65]. Gene models that picked up known LIM homeobox proteins by BLAST to the database of non-redundant proteins and contained LIM and homeobox domains were considered to be putative Lhx genes in these animals.

### Verification of gene models

Primers were designed using Primer3 http://frodo.wi.mit.edu to amplify *Nematostella*, *Hydra *and *Trichoplax *Lhx genes using TaKaRa reagents (TaKaRa Bio Inc., Shiga, Japan) (Additional file 1, Tables S2-6). Cloning was performed using the Zero Blunt TOPO PCR Cloning Kit for Sequencing (Invitrogen, Carlsbad, USA) and minipreps were performed using the standard Qiagen (Valencia, USA) protocol. Sequence concordance was analyzed using Sequencher 4.5 (Gene Codes Corporation, Ann Arbor, USA) and sequenced cDNAs were BLASTed against the genome sequence for verification followed by a Conserved Domain Search to confirm Lhx gene identity [66].

Three of the four putative LIM homeobox predicted proteins in *Hydra *and one in *Nematostella *contained only one LIM domain, though all known LIM homeobox proteins have two N-terminal LIM domains, followed by the C-terminal homeobox (Table 1). Genomic regions 1 kb upstream of these predicted gene models were analyzed for LIM domains by translating in three frames. 5' RLM-RACE PCR was performed (Ambion FirstChoice RLM-RACE Kit) to verify gene model predictions for potential upstream LIM domains in *Hydra *(see Additional file 1, Table S6 for primers used). The predicted gene models were also analyzed by lowering the e-value threshold in conserved domain searches http://www.ncbi.nlm.nih.gov/Structure/cdd/cdd.shtml[66].

In *Trichoplax*, one scaffold contained conflicting and overlapping gene predictions of Lhx genes (Additional file 1, Table S8). Some of these models appeared to have atypical domain composition such as having two LIM domains without a homeobox, while some had overlapping spatial location or different gene model predictions for the same locus. To determine the accuracy of hypothetical proteins, primers were designed to amplify all the predicted gene models by RT-PCR (Additional file 1, Table S3).

### Phylogenetic analyses and identification of introns

LIM homeobox gene sequences from *Nematostella*, *Hydra*, *Trichoplax *and *Amphimedon *were aligned with Lhx genes from other animals known to fall into different subfamilies using CLUSTALW [67,68]. The alignments were trimmed using GBlocks [69] and curated manually (both LIM domains and the homeodomain were used where available). Neighbor joining analyses were performed using Phylip [70] with default parameters and 500 bootstrap replicates. Maximum likelihoods were calculated using PhyML [71] with the WAG model of amino acid evolution, 4 substitution rate categories, proportion of invariable sites and γ distribution parameter estimated from the dataset, and 100 bootstrap replicates. Bayesian analyses were performed using MrBayes [72,73]; 2 chains were started and allowed to run for over 1 million generations, 1 tree was sampled every 100 generations, and the first 1,000 trees were discarded as burn-in. Orthologous Lhx genes from different species were aligned for each Lhx subfamily and conserved introns identified as described in Putnam *et al. *[48].

### Probe synthesis and *in situ *hybridization

Clones of *Nematostella*, *Hydra*, *Trichoplax*, and *Amphimedon *Lhx genes made using primers listed in Additional file 1, Tables S2-6 were used for probe synthesis. Digoxigenin (DIG)-labeled antisense and sense RNA probes corresponding to the putative Lhx genes in *Nematostella *were synthesized using labeling mix and T7/T3/Sp6 RNA polymerases from Roche Applied Science (Indianapolis, USA). *Nematostella *embryos at various stages were collected and fixed and *in situ *hybridization performed as described in Finnerty *et al. *[74]. DIG-labeled RNA probes were used at a concentration of 2 ng/μl for hybridization ranging from 12 to 48 h. *Amphimedon in situ *hybridizations were performed as described in Larroux *et al*. [64].

## Authors' contributions

MS and DSR designed the study on *Nematostella*, *Hydra *and *Trichoplax*. CL and BMD designed study of *Amphimedon *genes. MS cloned and studied expression of *Nematostella *genes and did the phylogenetic analyses. CL cloned and studied expression of *Amphimedon *genes. DRL and KM identified and cloned *Hydra *and *Trichoplax *genes respectively. JC identified the tandem Lhx cluster in *Trichoplax*. All authors contributed to the writing; MS and DSR organized the overall manuscript.

## Supplementary Material

Additional file 1 **Supplemental data**. Supplemental data including gene model sequences and domain summaries, primers used for amplifying Lhx orthologs, primers for 5' and 3' rapid amplification of cDNA ends (RACE), polymerase chain reaction (PCR) result summaries, ancestral linkage group assignments of human and *Trichoplax *Lhx genes, and detailed phylogenetic trees. Click here for file

## References

[B1] Greenspan RJ An introduction to nervous systems 2007 Cold Spring Harbor, New York: Cold Spring Harbor Laboratory Press

[B2] Srivastava M Begovic E Chapman J Putnam NH Hellsten U Kawashima T Kuo A Mitros T Salamov A Carpenter ML Signorovitch AY Moreno MA Kamm K Grimwood J Schmutz J Shapiro H Grigoriev IV Buss LW Schierwater B Dellaporta SL Rokhsar DS The Trichoplax genome and the nature of placozoans Nature 2008 454 955 960 10.1038/nature07191 18719581

[B3] Philippe H Derelle R Lopez P Pick K Borchiellini C Boury-Esnault N Vacelet J Renard E Houliston E Queinnec E Da Silva C Wincker P Le Guyader H Leys S Jackson DJ Schreiber F Erpenbeck D Morgenstern B Worheide G Manuel M Phylogenomics Revives Traditional Views on Deep Animal Relationships Curr Biol 2009 19345102 10.1016/j.cub.2009.02.052

[B4] Schierwater B Eitel M Jakob W Osigus HJ Hadrys H Dellaporta SL Kolokotronis SO Desalle R Concatenated analysis sheds light on early metazoan evolution and fuels a modern "urmetazoon" hypothesis PLoS Biol 2009 7 e20 10.1371/journal.pbio.1000020 19175291 PMC2631068

[B5] Sperling EA Peterson KJ Pisani D Phylogenetic-signal dissection of nuclear housekeeping genes supports the paraphyly of sponges and the monophyly of Eumetazoa Mol Biol Evol 2009 26 2261 2274 10.1093/molbev/msp148 19597161

[B6] Dunn CW Hejnol A Matus DQ Pang K Browne WE Smith SA Seaver E Rouse GW Obst M Edgecombe GD Sorensen MV Haddock SH Schmidt-Rhaesa A Okusu A Kristensen RM Wheeler WC Martindale MQ Giribet G Broad phylogenomic sampling improves resolution of the animal tree of life Nature 2008 452 745 749 10.1038/nature06614 18322464

[B7] Simpson TL The Cell Biology of Sponges 1984 New York: Springer-Verlag

[B8] Grell KG Ruthmann A Harrison FW Westfall JA Placozoa Placozoa, Porifera, Cnidaria and Ctenophora 1991 2 New York: Wiley-Liss 13 27

[B9] Lentz TL Primitive Nervous Systems 1968 New Haven: Yale University Press

[B10] Sakarya O Armstrong KA Adamska M Adamski M Wang IF Tidor B Degnan BM Oakley TH Kosik KS A post-synaptic scaffold at the origin of the animal kingdom PLoS ONE 2007 2 e506 10.1371/journal.pone.0000506 17551586 PMC1876816

[B11] Brusca RC Brusca GJ Invertebrates 2002 Sunderland, MA: Sinauer Associates

[B12] Gillis MA Anctil M Monoamine release by neurons of a primitive nervous system: an amperometric study J Neurochem 2001 76 1774 1784 10.1046/j.1471-4159.2001.00172.x 11259495

[B13] Grimmelikhuijzen CJ Williamson M Hansen GN Neuropeptides in cnidarians Can J Zool 2002 80 1690 1702 10.1139/z02-137

[B14] Kass-Simon G Pierobon P Cnidarian chemical neurotransmission, an updated overview Comp Biochem Physiol A Mol Integr Physiol 2007 146 9 25 10.1016/j.cbpa.2006.09.008 17101286

[B15] Westfall JA Neural pathways and innervation of cnidocytes in tentacles of sea anemones Hydrobiologia 2004 530/531 117 121 10.1007/s10750-004-2678-0

[B16] Hansen GN Williamson M Grimmelikhuijzen CJ Two-color double-labeling in situ hybridization of whole-mount Hydra using RNA probes for five different Hydra neuropeptide preprohormones: evidence for colocalization Cell Tissue Res 2000 301 245 253 10.1007/s004410000240 10955720

[B17] Hansen GN Williamson M Grimmelikhuijzen CJ A new case of neuropeptide coexpression (RGamide and LWamides) in Hydra, found by whole-mount, two-color double-labeling in situ hybridization Cell Tissue Res 2002 308 157 165 10.1007/s00441-002-0534-y 12012215

[B18] Hayakawa E Takahashi T Nishimiya-Fujisawa C Fujisawa T A novel neuropeptide (FRamide) family identified by a peptidomic approach in Hydra magnipapillata FEBS J 2007 274 5438 5448 10.1111/j.1742-4658.2007.06071.x 17894820

[B19] Darmer D Hauser F Nothacker HP Bosch TC Williamson M Grimmelikhuijzen CJ Three different prohormones yield a variety of Hydra-RFamide (Arg-Phe-NH2) neuropeptides in Hydra magnipapillata Biochem J 1998 332 2 403 412 9601069 10.1042/bj3320403 PMC1219495

[B20] Marlow HQ Srivastava M Matus DQ Rokhsar D Martindale MQ Anatomy and development of the nervous system of Nematostella vectensis, an anthozoan cnidarian Dev Neurobiol 2009 69 235 254 10.1002/dneu.20698 19170043

[B21] Curtiss J Heilig JS Delimiting development Bioessays 1998 20 58 69 10.1002/(SICI)1521-1878(199801)20:1<58::AID-BIES9>3.0.CO;2-O 9504048

[B22] Kadrmas JL Beckerle MC The LIM domain: from the cytoskeleton to the nucleus Nat Rev Mol Cell Biol 2004 5 920 931 10.1038/nrm1499 15520811

[B23] Hobert O Westphal H Functions of LIM-homeobox genes Trends Genet 2000 16 75 83 10.1016/S0168-9525(99)01883-1 10652534

[B24] Gehring WJ Affolter M Burglin T Homeodomain proteins Annu Rev Biochem 1994 63 487 526 10.1146/annurev.bi.63.070194.002415 7979246

[B25] Lin Y Zhao J Chen S Zeng X Du Q Yang Y Lu F Pu Y Yang Z A novel mutation in LMX1B gene causes nail-patella syndrome in a large Chinese family Bone 2008 43 591 595 10.1016/j.bone.2008.04.025 18595794

[B26] Krawchuk D Kania A Identification of genes controlled by LMX1B in the developing mouse limb bud Dev Dyn 2008 237 1183 1192 10.1002/dvdy.21514 18351676

[B27] Tosney KW Hotary KB Lance-Jones C Specifying the target identity of motoneurons Bioessays 1995 17 379 382 10.1002/bies.950170503 7786283

[B28] Gill GN Decoding the LIM development code Trans Am Clin Climatol Assoc 2003 114 179 189 12813919 PMC2194522

[B29] Dawid IB Chitnis AB Lim homeobox genes and the CNS: a close relationship Neuron 2001 30 301 303 10.1016/S0896-6273(01)00307-5 11394990

[B30] Cheng L Chen CL Luo P Tan M Qiu M Johnson R Ma Q Lmx1b, Pet-1, and Nkx2.2 coordinately specify serotonergic neurotransmitter phenotype J Neurosci 2003 23 9961 9967 14602809 10.1523/JNEUROSCI.23-31-09961.2003 PMC6740868

[B31] Ding YQ Marklund U Yuan W Yin J Wegman L Ericson J Deneris E Johnson RL Chen ZF Lmx1b is essential for the development of serotonergic neurons Nat Neurosci 2003 6 933 938 10.1038/nn1104 12897786

[B32] Simon HH Bhatt L Gherbassi D Sgado P Alberi L Midbrain dopaminergic neurons: determination of their developmental fate by transcription factors Ann N Y Acad Sci 2003 991 36 47 12846972

[B33] Zhao Y Marin O Hermesz E Powell A Flames N Palkovits M Rubenstein JL Westphal H The LIM-homeobox gene Lhx8 is required for the development of many cholinergic neurons in the mouse forebrain Proc Natl Acad Sci USA 2003 100 9005 9010 10.1073/pnas.1537759100 12855770 PMC166428

[B34] Elshatory Y Everhart D Deng M Xie X Barlow RB Gan L Islet-1 controls the differentiation of retinal bipolar and cholinergic amacrine cells J Neurosci 2007 27 12707 12720 10.1523/JNEUROSCI.3951-07.2007 18003851 PMC2972590

[B35] Elshatory Y Gan L The LIM-homeobox gene Islet-1 is required for the development of restricted forebrain cholinergic neurons J Neurosci 2008 28 3291 3297 10.1523/JNEUROSCI.5730-07.2008 18367596 PMC2786914

[B36] Manabe T Tatsumi K Inoue M Matsuyoshi H Makinodan M Yokoyama S Wanaka A L3/Lhx8 is involved in the determination of cholinergic or GABAergic cell fate J Neurochem 2005 94 723 730 10.1111/j.1471-4159.2005.03261.x 16000160

[B37] Manabe T Tatsumi K Inoue M Makinodan M Yamauchi T Makinodan E Yokoyama S Sakumura R Wanaka A L3/Lhx8 is a pivotal factor for cholinergic differentiation of murine embryonic stem cells Cell Death Differ 2007 14 1080 1085 10.1038/sj.cdd.4402106 17318222

[B38] Bachy I Retaux S GABAergic specification in the basal forebrain is controlled by the LIM-hd factor Lhx7 Dev Biol 2006 291 218 226 10.1016/j.ydbio.2005.10.023 16438949

[B39] Fogarty M Grist M Gelman D Marin O Pachnis V Kessaris N Spatial genetic patterning of the embryonic neuroepithelium generates GABAergic interneuron diversity in the adult cortex J Neurosci 2007 27 10935 10946 10.1523/JNEUROSCI.1629-07.2007 17928435 PMC6672847

[B40] Cassata G Kagoshima H Andachi Y Kohara Y Durrenberger MB Hall DH Burglin TR The LIM homeobox gene ceh-14 confers thermosensory function to the AFD neurons in Caenorhabditis elegans Neuron 2000 25 587 597 10.1016/S0896-6273(00)81062-4 10774727

[B41] Poche RA Kwan KM Raven MA Furuta Y Reese BE Behringer RR Lim1 is essential for the correct laminar positioning of retinal horizontal cells J Neurosci 2007 27 14099 14107 10.1523/JNEUROSCI.4046-07.2007 18094249 PMC6673498

[B42] Fischer AJ Foster S Scott MA Sherwood P Transient expression of LIM-domain transcription factors is coincident with delayed maturation of photoreceptors in the chicken retina J Comp Neurol 2008 506 584 603 10.1002/cne.21578 18072193 PMC2774723

[B43] Tsalik EL Niacaris T Wenick AS Pau K Avery L Hobert O LIM homeobox gene-dependent expression of biogenic amine receptors in restricted regions of the C. elegans nervous system Dev Biol 2003 263 81 102 10.1016/S0012-1606(03)00447-0 14568548 PMC4445141

[B44] Sarafi-Reinach TR Melkman T Hobert O Sengupta P The lin-11 LIM homeobox gene specifies olfactory and chemosensory neuron fates in C. elegans Development 2001 128 3269 3281 11546744 10.1242/dev.128.17.3269

[B45] Ryan JF Burton PM Mazza ME Kwong GK Mullikin JC Finnerty JR The cnidarian-bilaterian ancestor possessed at least 56 homeoboxes. Evidence from the starlet sea anemone, Nematostella vectensis Genome Biol 2006 7 R64 10.1186/gb-2006-7-7-r64 16867185 PMC1779571

[B46] Larroux C Fahey B Degnan SM Adamski M Rokhsar DS Degnan BM The NK homeobox gene cluster predates the origin of Hox genes Curr Biol 2007 17 706 710 10.1016/j.cub.2007.03.008 17379523

[B47] Larroux C Luke GN Koopman P Rokhsar DS Shimeld SM Degnan BM Genesis and expansion of metazoan transcription factor gene classes Mol Biol Evol 2008 25 980 996 10.1093/molbev/msn047 18296413

[B48] Putnam NH Srivastava M Hellsten U Dirks B Chapman J Salamov A Terry A Shapiro H Lindquist E Kapitonov VV Jurka J Genikhovich G Grigoriev IV Lucas SM Steele RE Finnerty JR Technau U Martindale MQ Rokhsar DS Sea anemone genome reveals ancestral eumetazoan gene repertoire and genomic organization Science 2007 317 86 94 10.1126/science.1139158 17615350

[B49] Chapman J Simakov O Rokhsar D David CN Steele RE The dynamic genome of *Hydra* Nature 2010 in press 10.1038/nature08830 PMC4479502 20228792

[B50] Peterson KJ Sperling EA Poriferan ANTP genes: primitively simple or secondarily reduced? Evol Dev 2007 9 405 408 17845512 10.1111/j.1525-142X.2007.00179.x

[B51] Putnam NH Butts T Ferrier DE Furlong RF Hellsten U Kawashima T Robinson-Rechavi M Shoguchi E Terry A Yu JK Benito-Gutierrez EL Dubchak I Garcia-Fernandez J Gibson-Brown JJ Grigoriev IV Horton AC de Jong PJ Jurka J Kapitonov VV Kohara Y Kuroki Y Lindquist E Lucas S Osoegawa K Pennacchio LA Salamov AA Satou Y Sauka-Spengler T Schmutz J Shin IT The amphioxus genome and the evolution of the chordate karyotype Nature 2008 453 1064 1071 10.1038/nature06967 18563158

[B52] Bürglin TR Duboule D A comprehensive classification of homeobox genes Guidebook to the Homeobox Genes 1994 New York: Oxford University Press 35 72

[B53] Marchler-Bauer A Anderson JB Derbyshire MK DeWeese-Scott C Gonzales NR Gwadz M Hao L He S Hurwitz DI Jackson JD Ke Z Krylov D Lanczycki CJ Liebert CA Liu C Lu F Lu S Marchler GH Mullokandov M Song JS Thanki N Yamashita RA Yin JJ Zhang D Bryant SH CDD: a conserved domain database for interactive domain family analysis Nucleic Acids Res 2007 35 D237 240 10.1093/nar/gkl951 17135202 PMC1751546

[B54] Matus DQ Magie CR Pang K Martindale MQ Thomsen GH The Hedgehog gene family of the cnidarian, Nematostella vectensis, and implications for understanding metazoan Hedgehog pathway evolution Dev Biol 2008 313 501 518 10.1016/j.ydbio.2007.09.032 18068698 PMC2288667

[B55] Adamska M Matus DQ Adamski M Green K Rokhsar DS Martindale MQ Degnan BM The evolutionary origin of hedgehog proteins Curr Biol 2007 17 R836 837 10.1016/j.cub.2007.08.010 17925209

[B56] Yasuoka Y Kobayashi M Kurokawa D Akasaka K Saiga H Taira M Evolutionary origins of blastoporal expression and organizer activity of the vertebrate gastrula organizer gene lhx1 and its ancient metazoan paralog lhx3 Development 2009 136 2005 2014 10.1242/dev.028530 19439497

[B57] Matus DQ Pang K Daly M Martindale MQ Expression of Pax gene family members in the anthozoan cnidarian, Nematostella vectensis Evol Dev 2007 9 25 38 17227364 10.1111/j.1525-142X.2006.00135.x

[B58] Jakob W Sagasser S Dellaporta S Holland P Kuhn K Schierwater B The Trox-2 Hox/ParaHox gene of Trichoplax (Placozoa) marks an epithelial boundary Dev Genes Evol 2004 214 170 175 10.1007/s00427-004-0390-8 14997392

[B59] Satou Y Imai KS Satoh N Early embryonic expression of a LIM-homeobox gene Cs-lhx3 is downstream of beta-catenin and responsible for the endoderm differentiation in Ciona savignyi embryos Development 2001 128 3559 3570 11566860 10.1242/dev.128.18.3559

[B60] Wang Y Zhang PJ Yasui K Saiga H Expression of Bblhx3, a LIM-homeobox gene, in the development of amphioxus Branchiostoma belcheri tsingtauense Mech Dev 2002 117 315 319 10.1016/S0925-4773(02)00197-1 12204277

[B61] Matus DQ Pang K Marlow H Dunn CW Thomsen GH Martindale MQ Molecular evidence for deep evolutionary roots of bilaterality in animal development Proc Natl Acad Sci USA 2006 103 11195 11200 10.1073/pnas.0601257103 16837574 PMC1544064

[B62] Richards GS Simionato E Perron M Adamska M Vervoort M Degnan BM Sponge genes provide new insight into the evolutionary origin of the neurogenic circuit Curr Biol 2008 18 1156 1161 10.1016/j.cub.2008.06.074 18674909

[B63] Fritzenwanker JH Technau U Induction of gametogenesis in the basal cnidarian Nematostella vectensis(Anthozoa) Dev Genes Evol 2002 212 99 103 10.1007/s00427-002-0214-7 11914942

[B64] Larroux C Fahey B Liubicich D Hinman VF Gauthier M Gongora M Green K Worheide G Leys SP Degnan BM Developmental expression of transcription factor genes in a demosponge: insights into the origin of metazoan multicellularity Evol Dev 2006 8 150 173 10.1111/j.1525-142X.2006.00086.x 16509894

[B65] King N Westbrook MJ Young SL Kuo A Abedin M Chapman J Fairclough S Hellsten U Isogai Y Letunic I Marr M Pincus D Putnam N Rokas A Wright KJ Zuzow R Dirks W Good M Goodstein D Lemons D Li W Lyons JB Morris A Nichols S Richter DJ Salamov A Sequencing JG Bork P Lim WA Manning G The genome of the choanoflagellate Monosiga brevicollis and the origin of metazoans Nature 2008 451 783 788 10.1038/nature06617 18273011 PMC2562698

[B66] Marchler-Bauer A Anderson JB Chitsaz F Derbyshire MK DeWeese-Scott C Fong JH Geer LY Geer RC Gonzales NR Gwadz M He S Hurwitz DI Jackson JD Ke Z Lanczycki CJ Liebert CA Liu C Lu F Lu S Marchler GH Mullokandov M Song JS Tasneem A Thanki N Yamashita RA Zhang D Zhang N Bryant SH CDD: specific functional annotation with the Conserved Domain Database Nucleic Acids Res 2009 37 D205 210 10.1093/nar/gkn845 18984618 PMC2686570

[B67] Higgins DG CLUSTAL V: multiple alignment of DNA and protein sequences Methods Mol Biol 1994 25 307 318 8004173 10.1385/0-89603-276-0:307

[B68] Thompson JD Higgins DG Gibson TJ CLUSTAL W: improving the sensitivity of progressive multiple sequence alignment through sequence weighting, position-specific gap penalties and weight matrix choice Nucleic Acids Res 1994 22 4673 4680 10.1093/nar/22.22.4673 7984417 PMC308517

[B69] Castresana J Selection of conserved blocks from multiple alignments for their use in phylogenetic analysis Molecular Biology and Evolution 2000 17 540 552 10742046 10.1093/oxfordjournals.molbev.a026334

[B70] Felsenstein J PHYLIP -- Phylogeny Inference Package (Version 3.2) Cladistics 1989 5 164 166

[B71] Guindon S Gascuel O A simple, fast, and accurate algorithm to estimate large phylogenies by maximum likelihood Syst Biol 2003 52 696 704 10.1080/10635150390235520 14530136

[B72] Huelsenbeck JP Ronquist F MRBAYES: Bayesian inference of phylogenetic trees Bioinformatics 2001 17 754 755 10.1093/bioinformatics/17.8.754 11524383

[B73] Ronquist F Huelsenbeck JP MrBayes 3: Bayesian phylogenetic inference under mixed models Bioinformatics 2003 19 1572 1574 10.1093/bioinformatics/btg180 12912839

[B74] Finnerty JR Pang K Burton P Paulson D Martindale MQ Origins of bilateral symmetry: Hox and dpp expression in a sea anemone Science 2004 304 1335 1337 10.1126/science.1091946 15131263

