# Supplementary material for: Early evolution of the LIM homeobox gene family
Source: BMC Biol. 2010 Jan 18;8:4. doi: 10.1186/1741-7007-8-4 (PMC2828406; doi:10.1186/1741-7007-8-4)
Supplement: Additional file 1 — Supplemental data. Supplemental data including gene model sequences and domain summaries, primers used for amplifying Lhx orthologs, primers for 5' and 3' rapid amplification of cDNA ends (RACE), polymerase chain reaction (PCR) result summaries, ancestral linkage group assignments of human and Trichoplax Lhx genes, and detailed phylogenetic trees. [file 1741-7007-8-4-S1.PDF]

Supplemental Information

Early evolution of the LIM homeobox gene family

Mansi Srivastava[1,2]*, Claire Larroux[3], Daniel R. Lu[1], Kareshma Mohanty[1], Jarrod Chapman[4], Bernard M. Degnan[3], Daniel S. Rokhsar[1,4]

1. Center for Integrative Genomics and Department of Molecular and Cell Biology, University of California, Berkeley, CA 94720, USA.
2. Currecnt address: Whitehead Institute for Biomedical Research, Cambridge, MA 02142, USA.
3. School of Biological Sciences, The University of Queensland, Brisbane QLD 4072, Australia.
4. Department of Energy Joint Genome Institute, Walnut Creek, CA 94598, USA.

* Corresponding Author

Email Addresses:

MS: mansi@wi.mit.edu
CL: c.larroux1@uq.edu.au
DRL: danrlu@gmail.com
KM: karishma.mohanty@gmail.com
JC: jarrodc@gmail.com
BMD: b.degnan@uq.edu.au
DSR: dsrokhsar@gmail.com

Section 1: *Nematostella* Lhx gene models

>NvLmx (95727) protein prediction
CTGCNEPIEDRFLMKVVDEAWHESCLQCCICRSQLSRSCFSKDRKLYCRTDYEKTFGHRTTIWHLSFAVC TDNVFEKSLCLSLPCCDVS
NNTMPTERDIKEEIKTEANSDSDDSSVHGDDDDDKKGPKRPRTILTSQQRK VFKSAFEISSKPCRKVREELSRETGLSVRVVQVWFQNQ
RAKVQK

>NvLmx (95727) gene model
TGCACTGGCTGCAACGAGCCTATCGAAGATCGCTTCCTCATGAAAGTCGTGGACGAAGCGTGGCATGAGAGCTGTCTCCAGTGT
TGTATTTGTCGTTCTCAGCTCTCGCGCTCTTGCTTTAGCAAGGACCGCAAACTCTACTGTCGGACAGACTACGAAAAAACTTTCG
GTCATAGAACTACCATTTGGCATTTGTCATTCGCCGTTTGCACTGACAACGTTTTTGAAAAAAGTTTATGTTTATCTTTGCCATGT
TGTGATGTTAGTAATAATACAATGCCGACCGAAAGGGACATCAAAGAAGAAATAAAAACCGAGGCAAACTCTGACTCGGACGA
TTCTTCGGTTCATGGAGACGATGATGACGATAAAAAAGGACCGAAAAGACCGCGAACAATCCTTACGAGTCAGCAACGTAAGGT
ATTCAAGTCAGCCTTCGAAATCAGCTCCAAACCCTGTAGAAAGGTTCGCGAGGAGTTGTCTCGCGAGACAGGCTTGAGTGTAAG
AGTGGTCCAAGTCTGGTTCCAGAATCAAAGAGCAAAGGTACAGAAA

>NvLhx1/5 (109857) protein prediction
MVQQCAGCQLPIADKFLLKVLDGVWHAQCVQCSDCKCPLTERCFSREGKLFCKTDFYRRYGTKCSGCDQGISPNDMVRRAKHLVFH
VDCFVCSYCKRQITTGDELYYIGDGSFICRDDYYHSHPTNLDDAIDEPKDLSYGLDEDLDAALASKRRGPRTTIKAKQLEALKSTFAAT
PKPSRNIREKLAQETGLNMRVIQVWFQNRRSKERRLKQ

>NvLhx1/5 (109857) gene model
ATGGTTCAGCAGTGCGCTGGATGTCAGCTCCCAATCGCAGATAAATTCTTATTGAAGGTTTTAGATGGTGTGTGGCACGCGCAAT
GTGTCCAATGTAGCGACTGCAAGTGCCCATTGACAGAGCGATGTTTCTCACGAGAAGGCAAACTCTTCTGCAAAACCGACTTCTA
CAGACGATACGGGACCAAGTGCTCAGGCTGTGATCAAGGGATCTCGCCAAACGATATGGTTCGTCGAGCAAAACACTTGGTTTT
CCACGTTGACTGTTTTGTCTGTTCCTACTGCAAGCGCCAAATAACAACAGGCGACGAGCTATACTACATCGGAGATGGCAGTTTC
ATCTGTAGGGACGATTATTATCATTCCCATCCGACCAACCTTGATGATGCCATAGATGAGCCAAAGGATCTAAGTTACGGGTTGG
ACGAGGATTTGGACGCCGCACTGGCGAGCAAGCGACGTGGTCCGCGAACAACTATTAAAGCCAAGCAGCTAGAGGCCTTAAAA
TCTACTTTTGCGGCAACTCCTAAGCCGTCCAGAAACATTCGAGAAAAACTCGCTCAGGAAACGGGTCTAAATATGCGCGTAATTC
AAGTGTGGTTTCAAAATAGAAGGTCTAAAGAGAGGCGATTGAAGCAA

>NvIslet (161808) protein prediction
MPICCSFSAEKRRVSMCVGCGSQIHDQYILRVAPDLEWHASCLKCADCHMYLDEKCTCFVREGKTYCKRDYVRLFGTKCAKCSLNFS
KNDFVMRARNKIYHIDCFRCVACSRQLVPGDEFALREDGLFCKADHEIVEKATATAQATHVRNNGQRSSQSGGQTNGQTTPDSTPSK
RKTDRPTRVRTVLNEKQLHTLRTCYNANPRPDAMMKEQLVEMTGLSPRVIRVWFQNKRCKDKKKNPNSLSPTGAPPRTLPQQTLPAI
SGVPYGNSGSMRPDAMSDMSGYQPWKALNDFAMHSEMDQGAFQQLVHFSEQAPGSLPPSSDGIVSTGN*

>NvIslet (161808) gene model
ATGCCGATTTGTTGTTCTTTCTCTGCAGAGAAGCGGCGTGTGTCCATGTGTGTCGGCTGTGGATCTCAAATTCACGATCAGTACAT
CTTGAGGGTCGCGCCAGACCTTGAGTGGCATGCAAGTTGTCTAAAGTGTGCGGACTGTCATATGTATCTCGACGAGAAATGTACC
TGCTTCGTGCGTGAAGGGAAGACGTACTGCAAGCGAGACTACGTACGGTTATTCGGGACCAAGTGTGCGAAATGCTCGCTGAAC
TTCAGCAAGAACGATTTCGTTATGCGAGCGAGAAATAAAATCTACCACATCGATTGCTTTCGTTGCGTTGCGTGCAGCAGACAGC
TTGTGCCGGGGGACGAGTTCGCTCTTCGCGAAGATGGACTATTCTGTAAGGCCGATCACGAAATTGTAGAGAAAGCGACGGCGA
CGGCTCAGGCAACTCACGTACGGAACAACGGCCAGCGTTCTTCTCAGTCAGGGGGGCAAACTAATGGGCAGACGACGCCGGATT
CGACTCCAAGTAAAAGGAAAACTGATCGGCCGACAAGGGTTCGAACTGTGCTAAATGAAAAGCAGCTTCATACGCTAAGGACTT
GTTACAATGCGAACCCCCGGCCGGACGCCATGATGAAAGAACAACTAGTGGAAATGACGGGTCTGAGTCCGCGAGTCATAAGA
GTCTGGTTTCAAAACAAGCGTTGCAAGGATAAGAAAAAGAACCCTAATAGTCTTTCGCCCACCGGAGCGCCACCTAGAACTCTA
CCACAGCAAACATTACCAGCTATTTCGGGTGTTCCGTATGGAAACTCGGGATCGATGAGGCCCGATGCTATGTCTGATATGTCAG
GTTACCAACCATGGAAAGCGCTAAATGATTTCGCGATGCATAGCGAGATGGACCAAGGCGCATTTCAGCAATTGGTTCACTTCTC
AGAGCAAGCACCGGGGAGTTTACCGCCCTCCTCTGATGGGATAGTATCGACGGGAAATTAA

>NvArrowhead (246590) protein prediction
MTTPCEDKAVTSPSLNAVDHVSTQGERCVGCEAKIVDRYLVKVSGRAWHTKCLKCCLCSDELGREATCYTKDGKIYCKADYARQFG
TKCARCGRSIHANDWVRRAKNCVYHLACFACDNCKRQLSTGEEFALKDGHVLCKLHYLEALDTSPAERDYQEVFSPDVDGDDSPNS
LKSGRHKAKRVRTTFTEDQLQILQANFNIDSNPDGQDLERIAQLTGLSKRVTQVWFQNSRARQKKYGTPLYSPGLNSSSAVQSAMSCA
ILRSASSVKMVKVLPRGPPRLEEIVGKKHIISKNGSHFSLQLNT*

>NvArrowhead (246590) gene model
ATGACCACCCCGTGTGAAGATAAAGCTGTGACGAGTCCCTCGCTCAACGCCGTGGATCATGTGTCGACGCAGGGCGAGAGATGT
GTTGGGTGCGAGGCGAAGATCGTGGATCGGTACCTTGTGAAAGTGAGTGGTCGAGCCTGGCACACCAAGTGTTTAAAGTGCTGT
CTCTGCTCCGATGAACTTGGACGCGAGGCAACTTGCTACACGAAAGATGGGAAGATATACTGCAAAGCGGACTACGCAAGGCA
ATTTGGTACAAAATGTGCGCGTTGCGGTCGCAGTATTCACGCTAACGACTGGGTCCGTCGAGCCAAGAACTGTGTCTATCATCTC
GCTTGCTTCGCTTGTGATAACTGCAAGAGGCAGCTCTCAACCGGCGAGGAATTTGCTCTAAAGGATGGTCATGTACTCTGCAAGC
TCCATTACCTAGAGGCGTTGGACACTTCACCCGCGGAAAGAGATTACCAAGAAGTATTCTCACCCGATGTTGATGGCGACGACT
CTCCGAATAGCCTGAAGAGCGGCCGACACAAGGCGAAGCGGGTCCGAACCACGTTCACAGAAGACCAACTACAGATACTTCAA
GCGAACTTTAACATCGACTCGAATCCCGACGGACAAGATCTCGAGAGAATAGCGCAACTTACGGGACTCAGCAAACGGGTCACG
CAAGTATGGTTCCAGAACTCGAGAGCGCGCCAAAAGAAATACGGGACGCCGCTCTACAGCCCTGGTTTGAATTCGTCATCAGCG
GTACAATCTGCAATGTCATGCGCAATTCTCCGTTCTGCTTCAAGTGTCAAAATGGTGAAAGTCCTCCCCCGAGGGCCACCTCGAT

TAGAAGAAATAGTAGGGAAAAAGCATATTATATCTAAAAATGGATCCCATTTTAGCTTACAATTGAACACATAG

>NvApterous (98128) protein prediction
MGDKQQAVCAGCGSRILERFYLMAVDQEWHADCLKCSDCELRLDNELTCFSKDGVILCREDYYRRFSVKKCSSCSQAISSKELVMRA
RDQVYHVNCFACDRCKRMLATGEYFGMRGIRIYCKEDYEELLREESRNPTKINSLSKGRPRKRRIATAIESITNLGYDLSDRPGELTTG
ADGRPKRVRTSFKHHQLRAMKTYFAMNHNPDAKDLKQLSQKTGLTKRVLQVWFQNARAKFRRTVCNTSQTPMSPSCD

>NvApterous (98128) gene model
ATGGGTGACAAGCAGCAGGCGGTATGCGCGGGCTGCGGGTCTCGAATCCTCGAGAGGTTCTACCTGATGGCCGTGGACCAGGAA
TGGCATGCAGACTGTCTCAAGTGCAGCGATTGTGAACTGAGGCTTGACAATGAGCTAACCTGTTTCTCGAAGGACGGGGTCATA
CTATGCCGTGAGGATTACTATAGACGGTTTTCAGTCAAGAAATGTTCGTCCTGCAGTCAAGCTATCTCAAGCAAAGAGCTCGTTA
TGCGAGCCCGCGATCAAGTCTACCATGTCAACTGCTTCGCTTGCGATCGCTGTAAGCGCATGCTCGCTACAGGGGAATACTTTGG
AATGCGTGGAATCAGGATCTATTGTAAAGAGGACTACGAGGAGTTACTGCGTGAGGAATCGCGAAATCCAACCAAAATAAACTC
GTTATCGAAAGGAAGACCTCGGAAACGGCGGATTGCGACGGCAATAGAAAGCATCACTAATCTAGGATACGACCTCTCTGACCG
ACCCGGCGAACTTACGACTGGTGCCGACGGCCGTCCGAAACGCGTCCGAACTTCATTCAAGCATCACCAGCTTCGTGCGATGAA
GACTTACTTTGCCATGAATCACAACCCGGACGCAAAAGACTTGAAACAGCTCTCGCAAAAGACCGGTCTGACAAAAAGAGTTTT
ACAGGTTTGGTTTCAGAACGCGAGGGCAAAATTTCGTAGAACTGTGTGCAATACGTCGCAGACGCCGATGTCCCCGTCATGTGA
T

>NvLhx3/4 (15442) protein prediction
CAGCDEQFGHADRFILQALDRHWSXCIRCTDCDESLDVKCYVREGRVFCPSDYFRRFGPKCASCNKDIQPSQMVHKVDSNVYHITCLS
CVTCQQQLETRDEFFLLEDGKVICRNDYGDRDEGSDSSRSKRARTFISNDQLAFLKVAYASSPKTTLRDRERIAKETGLDMRVVQVWF
QNRRAKDKRL

>NvLhx3/4 (15442) gene model
TGCGCAGGGTGCGACGAGCAGTTTGGTCACGCGGACCGCTTCATACTCCAAGCACTAGACCGCCACTGGTCACGTGCATCCGCT
GTACAGACTGCGACGAGAGTCTTGACGTCAAGTGTTACGTCAGAGAAGGAAGGGTGTTTTGCCCAAGTGATTACTTCAGGCGTT
TCGGTCCCAAGTGCGCTAGCTGTAACAAGGACATCCAGCCATCTCAGATGGTCCACAAAGTCGACTCGAATGTATACCACATCA
CGTGCTTATCTTGTGTCACGTGTCAGCAACAGCTGGAGACTAGGGACGAGTTTTTTTACTAGAGGATGGTAAAGTCATTTGTAG
GAACGACTACGGCGACAGGGATGAAGGGTCGGACTCCTCGAGAAGCAAGCGCGCCCGCACGTTCATCAGCAATGACCAGCTGG
CTTTCCTTAAAGTCGCCTACGCGTCGTCGCCCAAGACCACTCTTCGAGATCGGGAGCGCATCGCAAAGGAGACTGGGCTCGACA
TGCGAGTGGTGCAGGTCTGGTTTCAGAATCGCCGTGCTAAAGACAAGCGATTA

Section 2: *Trichoplax* Lhx gene models

Scaffold_2 in *Trichoplax* contained conflicting putative Lhx gene models in one locus. One of three options were possible: 1) 20649, 2) 21472, or 3) 53308, 20437 and 53309. Neither 20647 nor 21472 were amplified via PCR, whereas 53308 and 20437 were. 20437 was cloned to confirm that the predicted gene model was indeed being expressed. This favors scenario 3, where three Lhx genes belonging to three different Lhx classes are present in tandem.

>TaLhx1/5 (30033) protein prediction
MISCAGCQLPICDKFLLSVLDRKWHTKCVQCSQCKVQLSEKCFSRDGKLYCRNDFYRTYGTKCSGCGIGIPPNELVRRARDDVYHIKCL
KCAICGRQMSTGEQLYINQHNQYICQADYQNSISSTNTSLNDQSLTDDKEDDNSDYDEKEDETEDLLDNNNEDDLQADNDNESGNNCK
KRGPRTTIKTEQLEMLKNAFAITPKPTRLIRERLAQQTGLNMRVIQVWFQNRRSKERRVKQNACRAMFRHNYQPYLLPYRHGQLRYD
MKLPQYSDETCSSPSFVPMIGKSIYISI*

>TaLhx1/5 (30033) gene model
ATGATCTCATGTGCCGGTTGCCAATTGCCAATATGTGATAAATTTCTCCTCAGCGTTTTAGATCGCAAATGGCATACCAAATGTGT
TCAATGTTCACAATGTAAAGTGCAATTAAGTGAAAAATGCTTTAGTCGTGATGGCAAATTATACTGTCGAAATGATTTCTATAGAA
CGTATGGAACTAAATGCTCAGGTTGTGGTATCGGAATACCGCCTAATGAATTAGTACGCCGAGCACGAGATGATGTCTACCATAT
CAAGTGTTTAAAATGTGCAATTTGTGGTCGTCAAATGTCAACTGGCGAGCAACTTTATATTAACCAACATAATCAGTACATTTGCC
AAGCTGATTATCAAAATTCAATTTCTTCAACTAATACAAGCCTAAATGATCAATCGTTAACCGATGACAAGGAAGATGATAATTC
GGACTATGACGAGAAAGAAGATGAAACAGAAGATCTATTAGATAATAATAATGAAGATGATCTTCAAGCTGATAACGATAATGA
AAGTGGAAATAATTGTAAGAAACGTGGACCGAGAACAACGATTAAGACAGAGCAATTAGAAATGTTAAAAAATGCTTTTGCTAT
AACGCCTAAGCCAACTAGATTGATACGGGAACGCTTGGCGCAACAAACAGGATTAAATATGCGTGTTATCCAAGTTTGGTTTCAA
AATCGACGCTCAAAAGAGCGACGTGTTAAACAAAATGCATGCCGAGCCATGTTCCGCCATAATTATCAACCATATTTATTGCCGT
ATAGACATGGACAATTACGTTATGATATGAAATTACCTCAATATAGTGACGAAACATGCAGTAGTCCTTCCTTTGTGCCAATGATA
GGTAAGTCCATTTACATAAGTATATAA

>TaLhx2/9 (14836) protein prediction
CAGCGRSIMDQEYLGIQKTDKKWHIQCLRCYDCNEPLDKDQSCYVKQGNIFCRTDYFNRFGSIKCPTCNSGISPKEHVMKAREYAYHC
SCFICHTCNRLLKTGEEFAMRGCKLYCKEHFQSITSDHRHSHHSNKDNSGENHDSSNSNDITDEHGRSKRIRTSFKQPQLRTMKTYFALN
HNPDSKDLKQLSIKTGLNKRVLQVWFQNARAKYRRSI

>TaLhx2/9 (14836) gene model
TGTGCTGGCTGTGGTCGTAGTATCATGGATCAAGAATATCTGGGTATTCAAAAAACCGACAAAAAATGGCACATACAATGTTTGC
GATGCTATGACTGCAATGAACCATTAGATAAAGATCAAAGTTGCTATGTTAAGCAAGGCAACATCTTTTGTAGGACCGATTATTTC
AACCGTTTTGGTAGCATCAAATGTCCAACTTGTAATTCTGGTATATCACCTAAAGAACATGTTATGAAAGCAAGAGAGTATGCTTA
TCATTGTTCTTGCTTTATTTGCCACACATGTAATAGATTGTTAAAAACAGGCGAAGAATTTGCAATGAGAGGATGCAAATTATATT
GCAAAGAGCATTTTCAATCTATTACTAGCGATCATCGGCACTCACATCATTCTAATAAAGATAATTCAGGAGAAAATCATGATAG
CTCTAATTCAAATGACATTACTGATGAACATGGGAGATCGAAACGTATAAGAACATCATTTAAACAACCTCAGTTACGAACTATG
AAAACTTATTTTGCTCTTAATCATAACCCAGATAGTAAAGATCTAAAGCAATTATCAATAAAGACAGGATTAAACAAGCGTGTAT
TACAAGTTTGGTTTCAGAATGCCCGAGCTAAATATCGTCGATCAATA

>TaIsl (30944) protein prediction
MLILISFFECPDQLPFCSGCGGKINDRYILQVAPDMQYHAACLKCASCQQLLDEKETCFLRNGKPYCKSDFKMLFHNRCTKCNRIFEPSE
FIMRAKGNPYHIDCFRCHSCMRKLIPGDRYGVDTYILYCKEHYLNKMSSSSNHDTLQSTMADSDWQNSDNTDTKSQSTHAKSKQLTSR
KGMKGTRIRTVLNEKQLQTLRSYYASNPRPDSTVKEKLVELTGLNPRVIRVWFQNKRCKDKKIKAAGEQALEEEVVRIILQYTVSIC*

>TaIsl (30944) gene model
ATGCTCATTTTGATTTCTTTTTTTGAATGCCCAGATCAATTACCTTTTTGCTCTGGTTGTGGTGGCAAGATCAACGATCGCTACATC
TTGCAGGTTGCACCTGATATGCAATATCATGCAGCTTGCTTAAAATGCGCTTCGTGCCAGCAACTGTTAGATGAAAAAGAAACAT
GTTTCCTACGAAATGGCAAACCTTACTGCAAGTCAGATTTTAAAATGTTATTCCATAATCGTTGTACAAAGTGTAACCGAATATTT
GAACCAAGTGAATTTATCATGAGAGCCAAAGGCAATCCTTACCATATCGATTGCTTCCGATGTCATAGCTGTATGCGCAAGCTAAT
TCCTGGTGATCGATATGGCGTCGACACTTACATTTTATATTGTAAAGAGCATTACTTAAATAAAATGAGTAGTAGTAGCAATCATG
ATACCTTGCAATCAACGATGGCGGACAGCGATTGGCAAAATTCCGATAATACTGATACCAAGTCTCAGTCAACGCATGCCAAATC
GAAACAATTAACTTCACGGAAAGGTATGAAAGGAACAAGAATTCGAACGGTACTCAATGAGAAGCAATTGCAGACGCTTAGATC
GTATTATGCGAGTAATCCAAGGCCCGATTCAACAGTAAAAGAAAAATTGGTAGAATTAACAGGACTAAATCCTAGAGTTATACGC
GTTTGGTTTCAAAATAAAAGATGTAAAGATAAGAAAATAAAAGCAGCTGGAGAGCAAGCTCTAGAGGAAGAGGTAGTAAGAATA
ATCTTGCAATATACCGTCAGTATCTGCTGA

>TaLhx3/4 (53309) protein prediction
MAIGNIDYKVPDFVITALYTLLSTFKCSIFGNTVNCSSRGYCMQAQGVATRIRNILQRFKSCRKDLKLLELDSRRNELLCFKCQEEIRDRN
IYEMDNQLWHGKCISCIDCGKNLEGKCFVKEESIYCSMHYYRRFGRKCQGCNLGILPDEMVYRLHGSCYHINCLLCIVCSRQFKVGDK
YYISDEGKPICKEDYDVAIMCSDDFQLHHPNLKRPRTSITQQQLKMLNSVYRIKPRPSRITREMIATKLMEELTRLRILVRCVDTGCSLCT
EIYTFNIVTPNCRTEFTICSIVMFTTPITN*

>TaLhx3/4 (53309) gene model
ATGGCTATTGGTAACATAGATTATAAAGTTCCTGATTTTGTAATTACAGCATTATACACCCTGCTAAGCACTTTTAAATGCTCAATC
TTCGGAAATACTGTAAACTGTTCATCTAGGGGTTATTGCATGCAAGCACAGGGAGTAGCAACTAGAATAAGAAATATACTTCAAA
GATTTAAGTCTTGCAGAAAAGATTTGAAGCTTTTGGAATTAGATAGTCGCCGAAATGAATTGCTGTGTTTTAAATGCCAAGAAGA
AATTCGCGATAGGAACATTTACGAAATGGATAATCAATTATGGCACGGCAAATGCATTTCATGTATAGATTGCGGTAAAAACCTC
GAAGGAAAATGTTTTGTAAAGGAAGAGTCAATCTATTGCAGCATGCATTACTACAGACGATTTGGACGTAAATGTCAAGGTTGCA
ATCTTGGTATCTTACCGGATGAGATGGTTTACCGACTTCATGGCTCTTGCTATCATATTAACTGCCTCCTTTGTATAGTATGTAGTA
GACAATTTAAAGTAGGCGATAAATATTACATTTCGGATGAAGGTAAACCAATTTGTAAAGAAGATTATGATGTCGCCATAATGTG
TAGCGATGATTTTCAGCTACATCATCCCAATCTTAAACGACCTCGAACATCTATAACACAACAGCAGTTAAAAATGCTTAATTCGG
TCTATCGAATTAAACCGAGACCAAGTAGAATTACCAGAGAAATGATTGCTACCAAGTTAATGGAAGAATTAACCAGACTTAGAAT
ATTGGTTCGATGTGTTGATACTGGATGCAGTCTTTGTACTGAGATCTATACTTTTAATATTGTTACCCCTAATTGCCGTACCGAATT
TACTATATGCTCAATTGTTATGTTTACGACTCCCATCACTAATTAG

>TaLmx (53308) protein prediction
MTSTMASVDNRTAMPHSLLAIKQKCTGCNQLIQDKFLLKVADDLWHEDCLRCYKCTQPLSKSCYIKDHKLYCKEDYDNNDYKVLRK
VHNGYVMVTNSLLDVDMHTQTSNRLLMTRLFGTRCDGCGRIIASKQELVMKALKKVYHVTCFFCQICKRQLKRGDEYILKDTKLYCR
ADYDKITNPSFANVAWNEEDIVNADLSLAILEEEEEEEEEEGSPSMDNKRKTDSYEDQSNNAKRQRTVLNPQQRKLFHDSFEKSSKPGK
EVRDELSRKTGLSARVVQVWFQNQRAKLKKENQETKGTRSHKGYRKYSYRDENSSQSDMKDSDSSDAGEYSGNKQD*

>TaLmx (53308) gene model
ATGACTTCTACTATGGCATCAGTTGACAATCGTACTGCTATGCCACACTCTTTATTAGCAATTAAGCAGAAGTGCACTGGCTGCAA
TCAACTCATTCAAGATAAATTTCTATTAAAAGTAGCCGACGATTTATGGCATGAAGATTGCCTAAGATGTTATAAATGTACTCAGC
CGTTGTCTAAAAGTTGTTATATCAAGGATCACAAGTTATACTGTAAAGAAGACTACGATAATAATGATTATAAAGTATTGAGAAA
GGTACATAATGGCTATGTCATGGTAACTAACTCACTTCTAGATGTTGATATGCATACACAGACATCGAATAGACTTCTTATGACGA
GGCTGTTTGGTACTCGATGCGATGGCTGTGGTCGCATTATTGCTTCGAAGCAAGAATTAGTCATGAAAGCTCTCAAGAAAGTTTAT
CATGTTACATGTTTTTTCTGCCAAATTTGTAAACGACAACTTAAGCGTGGTGATGAATATATATTAAAAGATACAAAGTTGTATTG
TAGAGCTGATTATGACAAAATTACCAATCCCTCCTTTGCTAACGTAGCATGGAATGAAGAAGACATAGTAAATGCTGATTTATCG
CTGGCTATATTAGAAGAAGAAGAAGAAGAAGAAGAGGAAGAAGGTTCTCCATCAATGGATAATAAAAGGAAAACAGATAGTTA
CGAAGACCAATCCAATAATGCCAAAAGGCAAAGAACTGTATTAAACCCTCAACAGAGAAAATTATTCCATGATTCTTTTGAGAAA
AGTTCTAAACCTGGCAAAGAGGTTAGAGATGAATTATCACGTAAAACAGGACTAAGCGCTCGAGTAGTTCAAGTCTGGTTTCAAA
ATCAACGAGCAAAATTGAAAAAAGAAAATCAGGAAACGAAAGGAACTAGAAGTCATAAAGGATATCGAAAATATTCTTATCGTG
ATGAAAATTCCAGTCAAAGTGACATGAAAGATTCCGATAGCTCAGACGCTGGAGAATATTCAGGAAATAAACAAGACTAG

>TaArrowhead (20437) protein prediction
CSACHQVIYDRHIWRVDNCSWHPNCLKCFKCNMSLQGSTSCFIKNNHLYCKSDYFKEFGVKCSRCKRYITSDDWIRRAKDNVYHLACF
ACDDCKRQLSTGEEFVLKNNRLLCRLHYVEQENSGEESIPLE*

>TaArrowhead (20437) gene model
TGTTCAGCTTGTCATCAAGTGATTTATGATCGTCATATTTGGCGTGTAGATAATTGTTCTTGGCATCCTAATTGCCTAAAGTGTTTC
AAATGTAATATGTCACTTCAAGGATCTACCTCATGCTTCATCAAAAACAATCATCTGTATTGCAAATCCGACTATTTTAAGGAGTT
TGGCGTTAAATGCTCTCGCTGTAAACGTTACATAACATCGGATGATTGGATTAGGCGCGCTAAAGATAATGTGTACCATTTGGCTT
GCTTCGCTTGCGATGATTGCAAACGGCAACTATCAACTGGTGAAGAATTTGTTTTGAAAAATAATCGTTTGTTATGTCGATTACAT
TATGTCGAACAAGAAAATTCGGGCGAAGAGAGTATTCCATTAGAATAG

Section 3: *Hydra* Lhx gene models

There is currently no gene model predicted for the Hydra Lhx1/5 gene in the JGI Hydra genome, although there is a model predicted in the NCBI database. Thus, NCBI reference numbers were used for the Hydra Lhx1/5 ortholog.

The region in bold letters in the *Hydra* Apterous gene model denotes revisions to the current Hydra gene model (Hma2.205679) as sequenced after performing 5' RACE PCR, and the corresponding amino acid corrections are shown by the region bold letters in the protein prediction. The region in italicized letters in the *Hydra* Apterous gene model denotes the 5' UTR that was also sequenced.

5' RACE PCR confirmed that the gene model for the Hydra Arrowhead ortholog does not contain additional LIM domains apart from the LIM domain detected in the current gene model (Hma2.218130).

>HmLmx (206323) protein prediction
MMQTSFLCHECKSPILDSFLLNIGGNEYWHECCLKCHECCEKLFRVCYKKDGGVYCKYDYKRLFGIKCVACNNIITPNSIAMKVLDTNL
FHIDCFKCQNCGKMLEKGEEYAFKNQKLLCKADFNTEHICDNDETKHDGDDMKHDGDDIKHDADVFSSSDDSDSMSTTSPSSYEKNS
GYKRPRTILTTQQRQNFKTAFEQAPKPCRKIREKLSKETGLSVRVVQVWFQNQRAKLKKLHRKEENEKKLDIYKCNTGKLNSKKSKSP
SPKKFSSNEFNKITSLVGANDQPSSPYLQSREIKNNKKTIQPFLHQKSPIFTVPDCDTVRHQSKSCETTMHQPKNATNFKYTYEHSVKSTA
YTPVHNNTVITYTTGQISNIGNNFDESIMTNNNVSVTRNYDTYPINNASSMNQLYSKHVNYPLVNQCHLMPHLINTYL

>HmLmx (206323) gene model
atgatgcaaacttctttttatgccacgaatgcaaaagtccaatcttagatagcttcttgttaaatattggtggcaatgaatattggcatgaatgttgcctaaaatgccacgaatgttgtgaaaaacttttccgagtatgttacaa
aaaagatggtggtgtatactgcaagtacgattataaacgcttatttggtataaaatgtgtggcgtgtaacaacatcattacacccaattcaattgccatgaaagtgcttgatacgaacttgtttcacatagattgtttcaagtgt
caaaattgtgggaaaatgttagaaaaaggcgaagagtatgcttttaaaaatcaaaaacttttatgtaaagctgattttaatacggagcatatttgtgataatgacgaaacaaaacatgatggtgacgacatgaaacatgatg
gtgacgacataaaacatgatgctgacgtatttagtagcagcgatgattcagatagcatgtcaactacaagcccatctagttacgagaaaaacagtggttataaaagaccgagaacaattttaactacacagcaaagaca
aaactttaaaacagcttttgagcaagctccaaaaccttgccgtaagatacgtgaaaaactatccaaagaaaccggtctaagtgttcgtgttgttcaagtttggtttcaaaatcaaagagcaaagctcaaaaaacttcatcgt
aaagaggaaaacgaaaaaaaattagacatttataaatgcaatacaggaaaactaaactcaaaaaagagcaaatcgccatcaccaaagaaattttcttcaaatgagttcaataaaataaccagtttagttggtgcaaacga
ccaaccaagctctccgtatttgcaatctcgggaaattaaaaacaataaaaaaacaatacaaccatttcttcatcaaaaatcacctatatttacagtacctgattgtgatacagttcggcatcaatcaaaaagttgtgaaacaa
ctatgcatcaaccaaaaaatgcaacaaatttcaaatatacgtatgaacatagtgtcaagtctaccgcatatacccctgtacataataacacggttataacatataccactggacaaatttctaatattggaaacaactttgatg
aaagtataatgaccaacaataatgtttcagtgacaagaaattatgatacttatcctataaacaatgcttcatcaatgaatcaattatattcgaaacacgtaaattatcctttagtcaatcaatgtcatttaatgccgcatctaatca
acacatacctgtaa

>HmLhx1/5 (NCBI:221121866|ref|XP_002155267.1) protein prediction
MEKEHSSSKGDFNLNNVCSKCSLPMNDKFVAKILDMIMHEDCIRCFSCNCMLKDTCYFRESKFFCKDHFFRFYGVKCVACKKRIDPLN
MVHKYKENLIHVACLRCCFCNREATTGEEIHITENFTFVCKEDFEKYQNCSEIRNFISDESLEKSSNASVEPSDNEDEINKSRGPRTVINLK
QLEVLKETFKVNQKPSRLEREKLATKTGLTTRVIQVWFQNKRSKERRFRRQSLYENRLKVAMCYEFFKAESRNKATSTQEFNQPTLTCI
NDNSVMRLNDNNIWEENGLIANRLKRNLEKDEQICAFHRYTFGIAWSPPKTCFHPHHLNIKGKKSPALRASPIHVVISMSKRYNEVFSV
GAILCFTHLKQETAVSRVNENTNLCTETQTQQGQTYMTPATPDEEFDPGEINVSQEILDESLRSRESKKFAEAVAPGQEEILIDFLNSQNV
DEINSPLPIEIIRAQKVYKQSDSMGKLVILSLLDHTKYTKKFIMNTFECTKHRIETARKWHTSHKGFSFPEKKVFVRSSLDQTKCEHFLDFI
FTSGILHDVAYGITKLKYDSGEEQKIVHAILTTKYSHAIMFYRKSCSENNYIPLSDSSLWKVLHAIKPSSRKSLAELDDVTASGMNGFQTL
QKLAQRFSSKSLEAALEKGKRYLKTSYQTNCSVNDSNISSHSSKHALSDPSEKNLQSNTEISEVVCADCYDLCKAIEMIKELTIQNSDDA
DSIYDLEIAVKDVFNYIKHLMRDSQQKKAKIEAFKQLNDELLSGLKIFVKKFFRFDTERAKESILARKE

>HmLhx1/5 (NCBI:221121865|ref|XM_002155231.1) gene model
ATGGAAAAAGAACACAGTTCAAGTAAAGGAGATTTCAATTTAAATAATGTATGCTCAAAATGCTCATTGCCTATGAACGACAAGT
TTGTAGCAAAAATATTGGACATGATAATGCACGAAGATTGTATTAGATGTTTCAGTTGCAACTGTATGCTGAAGGACACTTGTTAT
TTTCGTGAGTCAAAATTCTTTTGCAAAGATCATTTTTTTAGGTTCTATGGCGTTAAATGTGTGGCTTGTAAAAAAAGAATCGATCCT
TTAAATATGGTGCACAAGTATAAAGAAAATTTAATCCACGTGGCATGTCTTCGGTGTTGTTTTTGTAACAGAGAAGCGACAACCG
GCGAAGAAATACATATTACAGAAAATTTTACTTTTGTGTGCAAAGAAGATTTTGAAAAATATCAAAACTGTTCAGAAATTAGAAA
TTTCATTTCTGACGAGTCTTTAGAAAAATCTTCCAACGCTTCCGTCGAACCAAGTGATAATGAAGATGAAATAAATAAAAGTCGA

GGTCCACGTACAGTAATTAATTTAAAACAACTCGAAGTGTTAAAAGAAACATTCAAAGTAAATCAAAAGCCATCTCGTCTTGAAA
GAGAAAAATTAGCAACTAAAACAGGTTTGACAACTCGAGTTATACAAGTTTGGTTTCAAAACAAAAGATCAAAAGAACGTCGATT
TAGACGTCAATCATTGTACGAAAATCGTCTTAAAGTAGCCATGTGCTATGAATTTTTTAAAGCTGAAAGTCGCAATAAAGCTACTT
CAACACAAGAATTTAATCAACCTACATTGACTTGCATCAACGACAACTCTGTCATGCGATTAAACGATAATAATATTTGGGAAGA
AAATGGACTTATTGCAAATAGACTTAAAAGAAATCTTGAAAAGGATGAACAAATATGTGCTTTTCACCGGTACACGTTTGGTATT
GCATGGAGTCCTCCAAAAACATGCTTTCATCCTCACCATCTAAATATAAAAGGAAAAAAATCTCCCGCTTTAAGGGCGTCACCAA
TACATGTCGTCATATCAATGTCAAAGCGATACAATGAAGTATTTTCAGTTGGTGCAATTCTGTGTTTTACCCATTTAAAGCAAGAA
ACTGCTGTATCTAGAGTCAACGAAAACACCAATTTATGTACAGAAACACAAACACAACAAGGACAAACATATATGACACCTGCTA
CTCCAGATGAAGAATTTGATCCCGGTGAAATTAATGTTTCACAGGAGATTTTGGACGAATCTCTAAGAAGCCGTGAGAGTAAAAA
ATTTGCGGAAGCTGTTGCTCCTGGACAAGAGGAAATACTTATAGATTTTCTTAACAGTCAAAATGTTGATGAAATAAATAGCCCTC
TCCCAATTGAAATTATTCGTGCTCAGAAAGTATATAAGCAAAGTGATTCCATGGGAAAGTTAGTTATCCTCTCATTATTAGACCAT
ACCAAGTATACCAAAAAGTTTATAATGAACACATTTGAGTGTACCAAACATCGTATAGAAACAGCAAGGAAATGGCATACATCCC
ATAAAGGGTTCTCATTTCCAGAGAAGAAAGTATTCGTCCGATCTAGTCTTGATCAAACAAAGTGTGAACATTTCTTAGACTTTATA
TTTACAAGCGGTATTTTGCATGATGTTGCTTATGGAATAACCAAATTAAAATACGACAGCGGTGAAGAACAAAAGATAGTGCATG
CAATACTGACGACAAAATATAGCCACGCTATCATGTTCTATCGAAAAAGTTGTAGTGAAAACAATTATATACCATTATCTGATTCA
AGTTTGTGGAAAGTATTGCATGAATAAAACCTTCAAGTCGAAAAAGCTTAGCTGAATTAGATGATGTTACTGCTTCAGGCATGAA
TGGTTTTCAAACATTACAAAAATTGGCACAAAGATTTAGTTCTAAATCTCTCGAAGCTGCCCTTGAAAAAGGAAAAAGGTATTTG
AAAACAAGTTATCAAACCAATTGTAGTGTCAATGACTCAAACATTTCTTCTCATAGCTCAAAACATGCCTTATCAGATCCATCTGA
AAAAAATCTTCAATCCAACACAGAGATATCAGAAGTGGTATGTGCCGATTGCTATGATTTGTGCAAAGCTATCGAGATGATCAAA
GAACTAACAATTCAAAATTCTGACGATGCTGATTCTATTTATGATTTGGAAATTGCTGTAAAGGATGTATTCAACTACATAAAACA
CCTGATGAGAGATTCTCAACAGAAAAAGGCAAAAATCGAAGCTTTTAAGCAATTAAACGATGAACTGCTTTCTGGCTTAAAGATT
TTTGTCAAAAAATTCTTCCGGTTTGATACAGAGAGGGCCAAAGAGAGTATTTTGGCAAGAAAGGAATGA

>HmApterous (205679 corrected model) protein prediction
MLNSSNLNLCDACHEIIYDRYISIXGNRKLHEECLQCSSCSVSVSLSGRCYEKNEQIFCEECYHLNFSLVQCKGCSQSIMKSEYVMK
TQENFYHVYCFRCNLCNSMLQSGERYGVKGDKLFCEAHYYGNNAIGMQQNNSNQDYFIGRSFEESSVILADVYSKTSFQQLASPSELE
MRKKKFQNRLMKEKRYRTTFTQEQVELMQKAFEIERNPDSASLQQLSKEINLSKRVLQVWFQNARAKHKKLHNEKPTTFEKPECYEN
YSELPTVEHSTLQINYCDHGDCDHSKVINDN

>HmApterous (205679 corrected model) gene model
GTGCGCGAGTGTTATACAGCGCCGTATTCTTTCTTCTGTTGACAAGCTTTTGAAGCTAATTAGGGGAGCTTTAAAGCTAATTTGCAAC
CTAATTAACACTACAACAATTTTAACAGGTTCTTGATTCTCAGTTTAAAAAAATCTTTTGATTTATTTAATTAAATCAGTTGGGTATCCT
GTTAAG**ATGCTTAACTCTTCAAATTTGAATCTATGTGACGCATGTCACGAAATCATATATGATCGTTACATTTCAATTGTNG**
GAAATAGAAAATTACACGAAGAATGTTTACAATGCTCTAGTTGTTCTGTTTCAGTGTCTTTGTCCGGACGTTGTTATGAAA
AAAATGAACAAATATTTTGCGAGGAATGCTATCATCTAAATTTTTCATTGGTCCAATGTAAAGGTTGTTCTCAAAGTATTATGA
AATCCGAGTACGTCATGAAAACTCAAGAAAACTTCTATCATGTTTATTGCTTTCGATGTAATTTATGTAATTCTATGTTGCAAAGT
GGAGAACGTTACGGTGTCAAAGGCGATAAGTTGTTTTGCGAAGCACATTACTACGGAAACAACGCTATTGGTATGCAACAGAATA
ATTCAAACCAAGATTACTTTATTGGAAGAAGCTTCGAAGAAAGTTCAGTAATTTTGGCAGATGTCTATTCCAAAACTTCCTTTCAA
CAATTAGCTTCACCAAGTGAACTCGAAATGCGCAAAAAAAAATTTCAAAACCGACTCATGAAAGAAAAAAGATACAGAACAACA
TTCACTCAAGAACAAGTAGAGTTGATGCAAAAAGCTTTTGAAATTGAAAGAAATCCAGACTCCGCATCATTGCAACAATTATCTA
AAGAAATAAACTTATCCAAAAGAGTTTTACAAGTGTGGTTTCAAAATGCAAGAGCCAAACATAAAAAGCTTCATAATGAAAAGC
CTACAACTTTTGAAAAACCAGAATGTTACGAAAACTATTCTGAACTACCTACAGTAGAACACAGCACATTGCAAATAAACTATTG
TGACCATGGCGATTGTGACCATTCTAAGGTTATAAATGATAATTAG

>HmArrowhead (218130) protein prediction
MHSTEISCEGNANLLKIKCSSCSRPIQLTDWIRRAKQNVYHLACFSCDICKRQLSTGEEFAFIEDSILCKLHYVEHLELPASCPVLLTNSSV
MENSPELIVDEDQMWIKHENKNKRVRTSFTDEQVLILQANFDLDANPDSNELERIAADVSLPKRVTQVWFQNSRARQKKQQQHKTRN
HYGHCGTCNYYPCSCSNSNSVSDK

>HmArrowhead (218130) gene model
atgcattcaactgaaataagttgtgagggtaatgctaatttattaaaaataaaatgttcttcttgctcaagaccaataca attaactgattggataagaagagctaagcaaaatgtgtatcatcttgcatgtttttcatgtgatat
atgtaagagacaactttctacaggtgaagaattcgcttttattgaagatagtatactatgcaaacttcattatgtggaacatcttgaactacctgcttcttgtcctgttttgttaactaatagttctgttatggaaaactctcctgaat
taattgttgatgaagatcaaatgtggataaagcatgaaaataaaaacaagagagttcgcacatcatttactgatgaacaggttctaattttacaagctaattttgatcttgatgcaaatcctgatagcaatgagctggaacgc
atagctgctgatgttagtcttccaaagcgtgtcactcaagtttggttccaaaattctagagcaagacaaaaaaagcagcagcagcataaaacaagaaatcattatggccattgtggaacttgcaattattatccgtgctcat
gctcgaattcaaacagtgtatcagataaatag

Table 1: Functions of Lhx gene families in bilaterians and expression in *Nematostella*. Functions specific to the nervous system are highlighted in red.

	Drosophila* and *C. elegans	**Vertebrates and protochordates**	***Nematostella* Expression**
**Lhx3/4 (Lim 3)**	Postmitotic neurons; motorneuron subtypes in overlap with islet; interneuron identity (axon projection); Ce: thermosensory AFD interneuron identity; Ce: spermatheca and hypodermis expression. Dm: expression in precursors of proventriculus (digestive system organ), dorsal vessel (heart field), alary muscles (associated with heart)	brain expression; motorneuron identity (axon projection); interneuron identity; hindbrain reticulospinal neuron identity (axon projection); pituitary organogenesis; retinal development; thymus development; lymphocytes; prochordates: endoderm differentiation during gastrulation, notochord, heart field (endoderm)	
**Lhx1/5 (Lin 11)**	brain expression; motorneuron and interneuron identity (axon projection); Ce: thermosensory AIZ, olfactory, chemosensory, touch sensory neuron identity (axon projection), neural migration; blastopore expression; Ce: vulva development, Dm: leg and antenna development	brain expression; motorneuron identity (axon projection); interneuron identity; hindbrain reticulospinal neuron identity (axon projection); neural migration; retinal development; cerebellum, hyppocampus, and hypothalamus (thermoregulatory) development; cell movement during gastrulation; blastoporal organizer; head organizer; neural induction; Mesenchyme-to-Epithelium Transitions; kidney and gonad development	**Pharyngeal endodermal ring** (GABAergic neurons)
**Lhx2/9 (Apterous)**	Development of nervous system; axon guidance; muscle and wing patterning; neurotransmitter choice	Brain and eye development; interneurons; hematopoiesis; limb development	**Pharyngeal ecto/endoderm Directive mesenteries** (adrenergic neurons)
**Lhx6/8 (Arrowhe ad)**	Neural expression; subset of motor and interneurons in brain; imaginal disc precursor tissues	Region-specific differentiation events in brain; first branchial arch	**Aboral ectoderm** (GABAergic neurons)
**Islet**	Motor and interneurons; axonal targeting; mesodermal cell types	Motor neuron development; pancreas formation; eye development (in fish)	**Planula endoderm Directive mesenteries** (adrenergic neurons)
**Lmx**	Post-mitotic neurons; GABA-ergic neurons; regulation of axonal trajectories; excretory organs	Interneurons; otic vescicle; kidney development	**Oral nerve ring Pharyngeal ectoderm Directive mesenateries** (adrenergic neurons)

Table 2: Primers used to amplify *Nematostella* Lhx orthologs

Model Number	Forward Primer	Reverse Primer
95727	GCAACGAGCCTATCGAAGAT	TGGAACCAGACTTGGACCAC
109857	AGCTCCCAATCGCAGATAAA	TGAAACCACACTTGAATTACGC
161808	ACATCTTGAGGGTCGCGCCAGA	TGACTCGCGGACTCAGACCCG
246590	GATCGTGGATCGGTACCTTG	GCGTCCCGTATTTCTTTTGG
98128	TCCTCGAGAGGTTCTACCTGA	CTCGCGTTCTGAAACCAAAC
15442	GACCGCTTCATACTCCAAGC	GTCTTTAGCACGGCGATTCT

Table 3: Primers used to amplify *Trichoplax* Lhx orthologs

Protein Models	Scaffold Number	Forward Primer	Reverse Primer
30944	15	CCTTTTTGCTCTGGTTGTGG	CTTGCTCTCCAGCTGCTTTT
30033	12	ATCGCAAATGGCATACCAAA	ATCATTGGCACAAAGGAAGG
53308	2	GCACTGGCTGCAATCAACT	GCGTCTGAGCTATCGGAATC
53309	2	CAGCATTATACACCCTGCTA	GTGATGGGAGTCGTAAACATA
14836	2	GCTGGCTGTGGTCGTAGTAT	GGGCATTCTGAAACCAAACT
20649	2	GAAGTGCACTGGCTGCAATC	CAGTTATGATTGAATGGGCTG
21472	2	GCACTGGCTGCAATCAACT	CAGTTATGATTGAATGGGCTG
20437	2	GATCGTCATATTTGGCGTGT	ACTCTCTTCGCCCGAATTTT
16471	2	GCACTGGCTGCAATCAACT	CAGTTATGATTGAATGGGCTGA

Table 4: Primers used to amplify *Hydra* Lhx orthologs

Primer	Sequence	Hydra magnipapillata Genome Location	Contig Exon Number
32355 Forward	GTGCGCGAGTGTTATACAGC	Contig32355:9319..9338	1
32355 Reverse	CAATCGCCATGGTCACAATA	Contig32355:20597..20578	4
37332 Forward	CTGATTGGATAAGAAGAGCT	Contig37332:84713..84732	1
37332 Reverse	TTGAATTCGAGCATGAGCAC	Contig37332:85219..85200	1
32814 Forward	TTTTATGCCACGAATGCAAA	Contig32814:14534..14553	1
32814 Reverse	GGTATATGCGGTAGACTTGA	Contig32814:33175..33156	6
38915 Forward	GCTCAAAATGCTCATTGCCTA	Contig38915:116080..116100	1
38915 Reverse	CAGAGTTGTCGTTGATGCAAG	Contig38915:133471..133451	5

Table 5: Primers used to amplify *Amphimedon* Lhx orthologs. For sequence details see Larroux et al. 2006.

***Amphimedon* Ortholog**	**Forward Primer**	**Reverse Primer**	**Range**	**Size**
AmqLhx1/5	ACGGTAGGCACTGACAAAGATCAGAGA	TCATTCAAACCCTGACCAACCAGTTCC	6-1086	1087
AmqLhx3/4	5' RACE primer	GTTTTGGGCTGACGCAGTAGG	1-900	900
AmqIsl	ATGGCTGTAAACTTTCAATAGAA	TTGAAACCATACTCTAATCACC	137-695	695

Table 6: Primers used for 3' and 5' RACE of *Hydra* Lhx orthologs

**5' RACE Primer**	**Sequence**	**Hydra magnipapillata Genome Location**	**Contig Exon Number**
32355 GSP 1 (outer)	TGCGGAGTCTGGATTTCTTT	Contig32355:20394..20375	4
32355 GSP 2 (inner)	ATCGCCTTTGACACCGTAAC	Contig32355:11547..11528	3
37332 GSP 1 (outer)	CTTGAGTGACACGCTTTGGA	Contig37332:85105..85086	1
37332 GSP 2 (inner)	GCTATGCGTTCCAGCTCATT	Contig37332:85070..85051	1

Table 7: *Nematostella* Lhx ortholog gene models

**Model Number**	**Genomic Location**	**Domain Content**	**Classification**	**Verified by PCR**
95727	40:424209-438987	L-H	Lmx	Yes
109857	97:533140-534046	L-L-H	Lhx5	Yes
161808	24:1574423-1580305	L-L-H	islet	Yes
246590	228:302206-310974	L-L-H	arrowhead	Yes
98128	48:56532-64464	L-L-H	apterous	Yes
15442	302:216117-219779	L-L-H	lhx3/4	No

Table 8: *Trichoplax* Lhx ortholog gene models

**Model Numbers**	**Genomic Location**	**Domain Content**	**Top Hits**	**Verified by PCR**
30944	15:1495474-1502480	LIM-LIM-HD (3 exons)	Islet	No
30033	12:1334218 - 1343778	LIM-LIM-HD (3 exons)	LHX 1/5	Yes
53308	2:3694166 - 3699749	LIM-LIM-HD (9 exons)	Lmx	No
20437	2:3705037 – 3707733	LIM-LIM (2 exons)	Arrowhead	Yes
53309	2:3716413 - 3719526	LIM-LIM-HD (6 exons)	LHX 3/4	No
16471	2:3724902 - 3727679	LIM-LIM (2 exons)	LIM domain only 4	Yes
14836	2:4699976 - 4705804	LIM-LIM-HD (3 exons)	Apterous	Yes
20649 (likely incorrect model)	2:3694880 - 3718801	LIM-LIM-HD (3 exons)	LHX 3	No
21472 (likely incorrect model)	2:3694874 - 3718801	LIM-LIM-HD (3 exons)	LHX 3/4	No

Table 9: *Hydra* Lhx ortholog gene models

Model Number	Genomic Location	Domain Content	Verified by PCR	Same as Predicted
Hma2.205679	Contig32355:9319..20628	LIM-LIM-HD (4 exons)	yes	no
Hma2.218130	Contig37332:84628..85239	LIM-HD (1 exon)	yes	yes
Hma2.206323	Contig32814:14518..33406	LIM-LIM-HD (6 exons)	yes	yes
NCBI	Contig38915:104634..150211	LIM-LIM-HD (6 exons)	yes	-

Table 10: Ancestral linkage groups (ACLG) for human and *Trichoplax* Lhx genes

Human gene	human chromosome position	ACLG #
lhx1	17: 32,368,612-32,374,596	12
lhx5	12: 112,385,077-112,394,260	12
isl2	15: 74,416,202-74,421,871	11
lmx1a	1: 163,437,729-163,592,576	10
lhx4	1: 178,466,065-178,510,811	10
lhx9	1: 196,148,244-196,168,344	10
lhx6	9: 124,004,677-124,031,041	10
lhx2	9: 125,813,710-125,835,257	10
lmx1b	9: 128,416,550-128,498,649	10
lhx3	9: 138,227,919-138,236,776	10
isl1	5: 50,714,715-50,726,308	8
lhx8	1: 75,366,707-75,399,806	8
***Trichoplax* gene**	***Trichoplax* scaffold position**	**ACLG #**
30944	15: 1495474 - 1502480	16
30033	12:1334218 - 1343778	15
14836	2:4699976-4705804	10
53308	2: 3694166 - 3699749	10
16471	2: 3724902 - 3727679	10
20437	2: 3705037 - 3707733	10
53309	2: 3716413 - 3719526	10

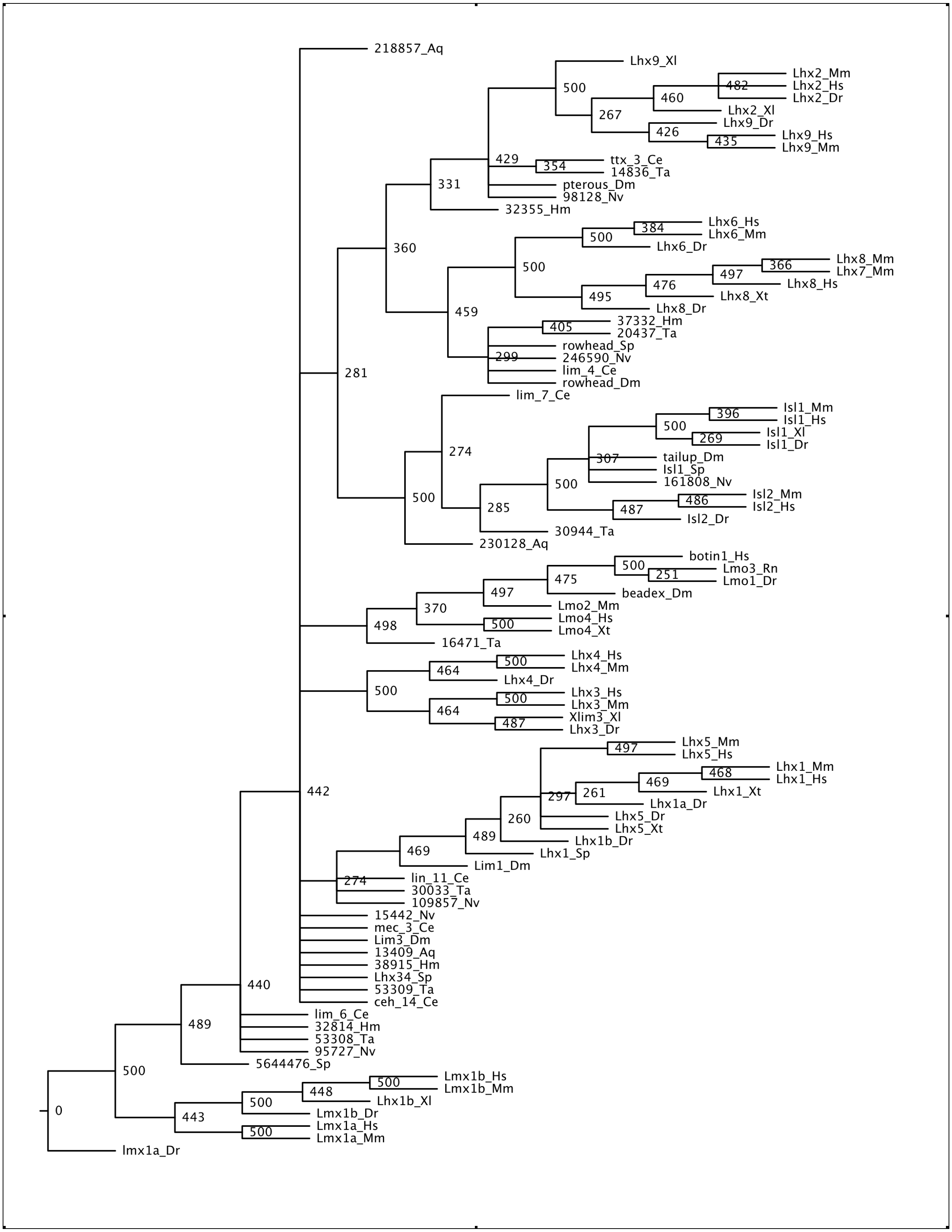

Figure 1: Unrooted neighbor joining tree with 500 bootstrap replicates for Lhx genes shown in Figure 1 of the main text.

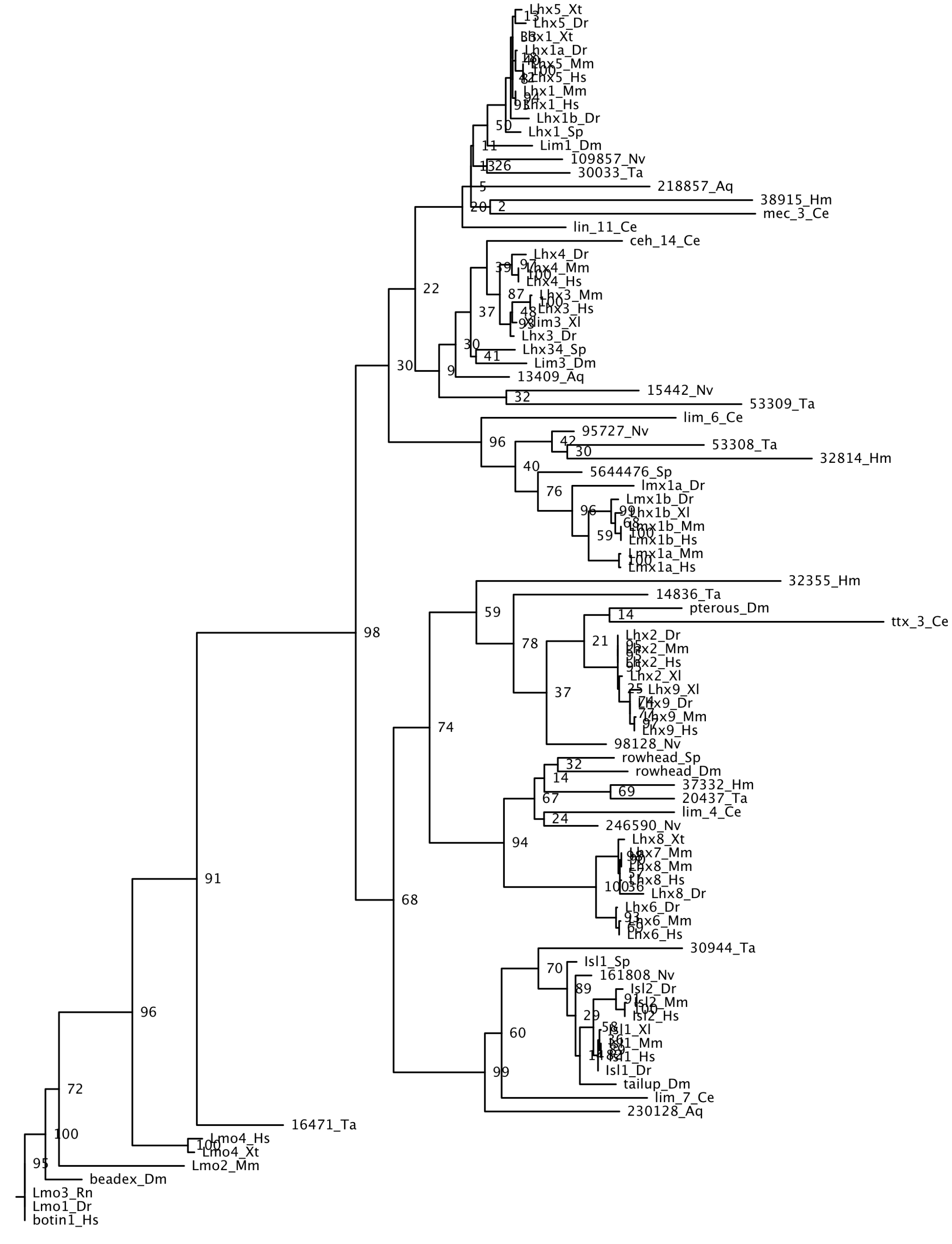

Figure 2: Unrooted maximum likelihood tree with 100 bootstrap replicates (using the WAG model of amino acid evolution, four substitution rate categories, proportion of invariable sites and gamma distribution parameter estimated from the dataset) for Lhx genes shown in Figure 1 of the main text.

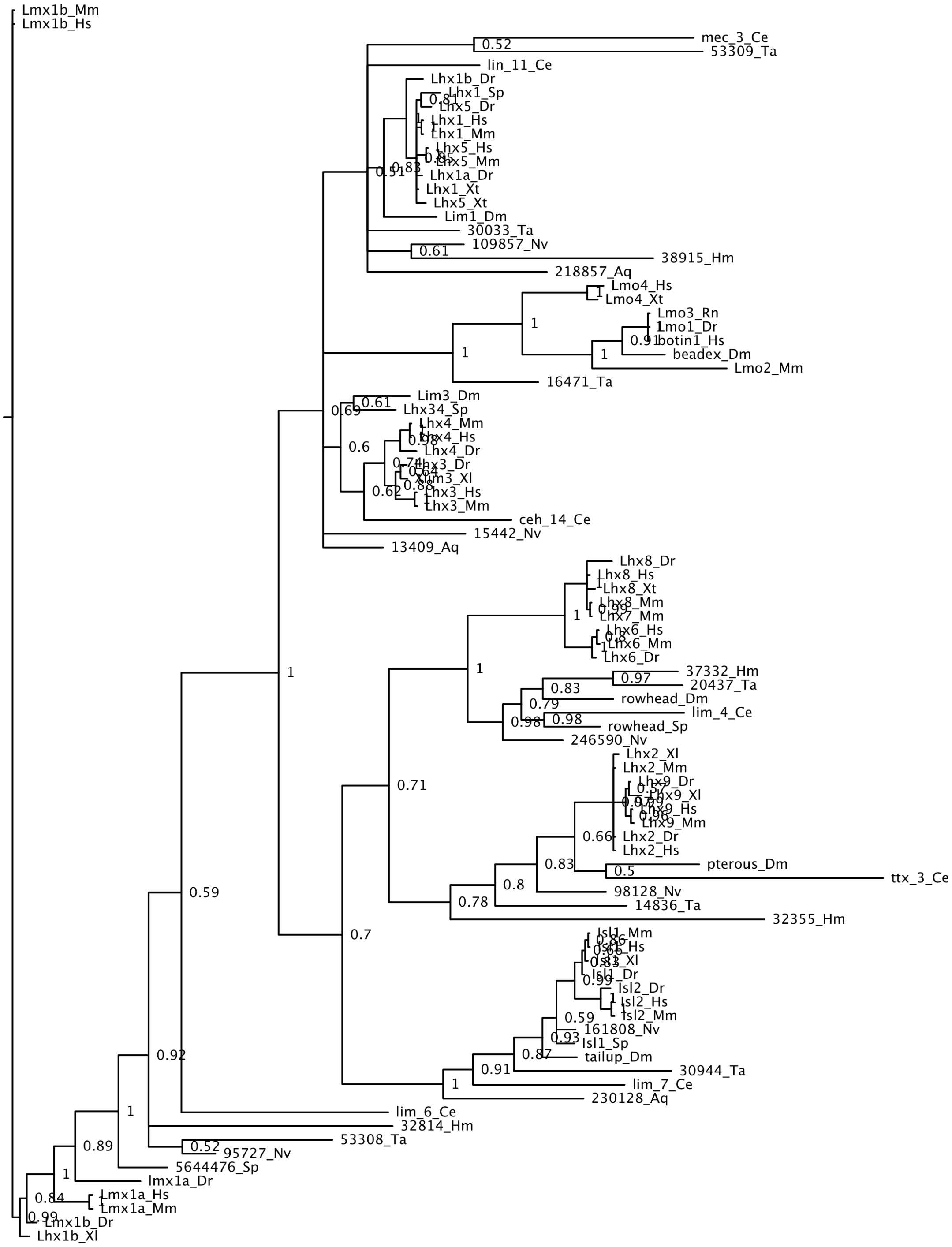

Figure 3: Unrooted Bayesian consensus tree after 1096100 generations (1 tree sampled every 100 generations, burnin = 1000 trees) for Lhx genes shown in Figure 1 of the main text.
